# A review on radiofrequency, laser, and microwave ablations and their thermal monitoring through fiber Bragg gratings

**DOI:** 10.1016/j.isci.2023.108260

**Published:** 2023-10-19

**Authors:** Elena De Vita, Daniela Lo Presti, Carlo Massaroni, Agostino Iadicicco, Emiliano Schena, Stefania Campopiano

**Affiliations:** 1 Department of Engineering, University of Naples “Parthenope”, 80143 Naples, Italy; 2 Department of Engineering, Università Campus Bio-Medico di Roma, 00128 Rome, Italy

**Keywords:** Optics, Materials science

## Abstract

Thermal ablation of tumors aims to apply extreme temperatures inside the target tissue to achieve substantial tumor destruction in a minimally invasive manner. Several techniques are comprised, classified according to the type of energy source. However, the lack of treatment selectivity still needs to be addressed, potentially causing two risks: i) incomplete tumor destruction and recurrence, or conversely, ii) damage of the surrounding healthy tissue. Therefore, the research herein reviewed seeks to develop sensing systems based on fiber Bragg gratings (FBGs) for thermal monitoring inside the lesion during radiofrequency, laser, and microwave ablation. This review shows that, mainly thanks to multiplexing and minimal invasiveness, FBGs provide an optimal sensing solution. Their temperature measurements are the feedback to control the ablation process and allow to investigate different treatments, compare their outcomes, and quantify the impact of factors such as proximity to thermal probe and blood vessels, perfusion, and tissue type.

## Graphical abstract

**Figure:**
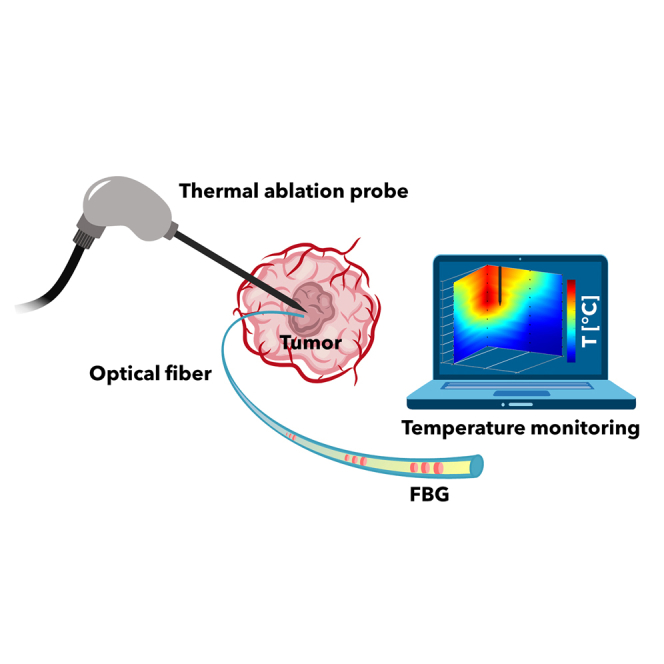

## Introduction

Cancer, among the first ten causes of death for almost all the countries in the world, is daunting in the breadth and scope of its diversity, spanning genetics, cell and tissue biology, pathology, and response to therapy.^1^ Since treatments and methods vary significantly depending on the cancer type and location, a general cure is really far from finding.^2^

Tumor detection, chemotherapy, and radiotherapy can prove insufficient to eradicate the tumor, involve dramatic side effects, and accelerate the growth of surviving tumor cells if not completed, respectively. Moreover, they often result inconvenient, due to the need of numerous treatments, associated with a delay in the onset of effect, and limited by dose. Excisional surgery is the mainstay treatment modality of solid tumors.^3^ It plays an important role in removing tumors from the body in many cases, and it is still efficient in terms of time, especially when combined with chemotherapy and imaging technology, such as X-ray, computed tomography (CT), magnetic resonance imaging (MRI), positron emission tomography (PET) and ultrasound (US).^4^ However, several factors can hamper its efficacy or even invalidate the surgical operation viability. Indeed, patient’s comorbidities, including respiratory and cardiac diseases, often preclude the use of anesthetics. Moreover, tumor morphologic features such as its presence in multiple anatomical sites, the unfavorable location, and other factors such as proximity to vital organs, encasement of major vessels or lesions deep to critical structures can contra-indicate invasive treatments. Furthermore, surgical resection can involve several adverse events, such as infection, wound dehiscence, hemorrhage, unbearable pain, esthetic deformities, and minimal residual disease, which is the occult neoplastic disease that remains *in situ* after curative surgery, resulting in tumor recurrence.^5^

Finally, depending on scale of the procedure and general health of the patient, recovery time and hospitalization costs must be considered, since some interventions may require post-operative admission to critical care.

Considering the invasive nature of surgical resection and the related severities, precision medicine in oncology field has gained momentum, leading to the development of innovative minimally invasive techniques, called tumor ablation, for treating tumors. The rise of this kind of therapy results in the minimization of collateral damages to healthy tissues, treatment pain, and hospitalization rate, and is based on the high accuracy introduced by robotic and/or image-guidance. Indeed, tumor ablation began to spread for treating primary and metastatic cancer in poor operative candidates thanks to the advances in laparoscopic surgical approach of the 70s and 80s and the significant evolution of imaging devices and modalities since the 90s.^6^ In general, ablative treatments are minimally invasive, well tolerated, and demonstrate a very low rate of major complications.^7^ Moreover, they allow the procedure the possibility to carry out further therapy to the same site with no treatment ceiling, unlike resection, chemo- or radiotherapy. Furthermore, ablation usually does not rule out the potential for future surgical resection, should this become feasible later.

Tumor ablation is defined as the direct application of thermal or chemical therapies to a specific focal tumor in order to achieve either eradication or substantial tumor destruction.^8^ More specifically, the term “direct” distinguishes tumor ablation therapies from others that are applied orally, intravascularly or through a peripheral venous route. Both chemical and thermal ablation exploit various physical, chemical, and biochemical properties of tumors to cause local cell necrosis.^6^

Chemical ablation employs chemical agents, such as absolute ethanol and acetic acid, relying on their toxicity to induce cell necrosis by intracellular dehydration and protein denaturation, followed by fibrosis and small-vessel thrombosis.^9^ It can be delivered intravenously, intra-arterially, or interstitially, by a rapid injection or a defined rate of infusion. Thermal ablation alters the local temperature of the tumoral cells which constitute the target tissue, either with heating or cooling mechanisms. Contrary to chemical ablation, which is classified based on employed agent, route, and delivery vehicle, thermal ablation techniques differ in the source of thermal energy involved to induce tissue necrosis and tumor destruction. An initial classification distinguishes between hyperthermal therapies, which heat the tumor at cytotoxic temperatures, and hypothermal therapies, known as cryoablation, involving the freezing of the target tissue. Then, among heat-based modalities, more therapeutical options are viable, i.e., radiofrequency ablation (RFA), laser ablation (LA), microwave ablation (MWA), and high-intensity focused ultrasound (HIFU). Depending on the type of technique, the involved energy source is normally applied via different applicators, i.e., electrodes for RFA, optical fibers for LA, antennas for MWA, transducers for HIFU, and cryoprobes in case of cryoablation.

It is important to highlight that for all the thermal ablation techniques, it is crucial to prevent the risk of an incomplete ablation, which in turn potentially leads to tumor recurrence, and against the involvement of the surrounding healthy tissue in the thermal treatment. The best way to ensure an effective and safe thermal ablation lies in temperature monitoring in such a way as to provide feedback on the treatment outcomes and control the ablation course. Therefore, this work focuses on thermal ablation, in particular on RFA, LA, and MWA techniques, aiming to deepen the role of temperature monitoring during this kind of treatments. The proposed solution for such thermal monitoring lies in fiber Bragg grating sensors (FBGs), a minimally invasive strategy not only for temperature measurements in real-time during the ablation procedure, but also to control the treatment outcomes. Section Thermal ablation techniques: Radiofrequency ablation, laser ablation, and microwave ablation in comparison describes their working principle and clinical application, with a view to compare them in terms of technology, heating mechanism, advantages, and disadvantages. Moreover, the criteria usually implemented by clinicians to choose the appropriate technique are discussed, reporting some examples of decisional algorithms. Section Temperature monitoring need raises the issue of treatment selectivity, introducing the dependence of tumor destruction completeness on several factors, first and foremost temperature gradient inside the lesion, and underlying the need of temperature monitoring for controlling the treatment success. Section Fiber optic sensors in thermal ablation outlines an overall framework of the main temperature monitoring techniques in thermal ablation field, from contactless approaches to invasive measurements moving toward fiber optic sensor, with a focus on FBG principles and features. The studies in literature based on the use of FBGs for thermal monitoring during RFA, LA, and MWA are reviewed in Section Results of thermal ablation controlled by fiber Bragg gratings, reporting the main achievements in terms of ablation implications which different configurations of FBGs allow investigating.

## Thermal ablation techniques: Radiofrequency ablation, laser ablation, and microwave ablation in comparison

Human cells can survive up to about 42°C.^10^ When the temperature is around 42÷45°C, cell damage occurs after prolonged exposure (from 30 to 60 min). This is the case of traditional hyperthermia, used as adjuvant to radiotherapy and chemotherapy to enhance the cancerous tissue sensitivity to such conventional therapies.^11^ By increasing the involved temperatures, different therapeutic results can be observed, since cytotoxicity and anti-tumor immunity vary with temperature. Therefore, besides traditional hyperthermia, much higher temperatures above 50°C can be involved, resulting in thermal ablation therapies, which can be used as stand-alone therapies.^12^ In this case, shorter times (from seconds to minutes) are enough for irreversibly damaging cells. Indeed, at temperatures ranging from 60°C to 140°C, protein denaturation occurs in few seconds, leading to coagulative necrosis almost immediately, where coagulation necrosis is intended as irreversible thermal damage of cells, even if the ultimate manifestations of cell death do not fulfill the strict histologic criteria of coagulative necrosis.^13^ Above 100°C, vaporization phenomenon of the water content of the target tissue prevails, followed by carbonization and smoke generation when increasingly higher temperatures are involved.^14^^,^^15^

During hyperthermal ablation treatments, the heat injury occurs in two subsequent phases: i) directly, due to the total thermal energy applied to the tumor, depending on its biology and microenvironment, and ii) indirectly, causing a progression in tissue damage after the cessation of the focal hyperthermal stimulus. Direct injury is generally better defined than the indirect effects, and occurs at several levels, from the subcellular level to the tissue level.^14^ Typically, thermal lesions are characterized by three concentric zones.^16^^,^^17^ The central one, immediately around the applicator tip of the thermal probe, is distinguished by its dark color due to the ablation-induced coagulative necrosis experienced by the corresponding tissue. The peripheral or transitional zone is where thermal conduction from the central area happens, causing a reversible injury. Finally, the tissue which surrounds the aforementioned regions remains unaffected by ablation. The full extent of tissue damage becomes evident from one to seven days after the thermal therapy; then, the lesion size decreases slightly during the following weeks because of the breakdown and removal of necrotic tissue by inflammatory cells.

For RFA, LA, and MWA implementation, two strategies can be adopted in the clinical practice, i.e., centrifugal coverage and centripetal convergent methods.^18^ The first involves a centrifugal energy dissipation toward the periphery from an applicator inserted into the center of the tumor target and it is the simplest and thus widely used method. It is suitable for the simultaneous and fast treatment of several small tumors. The second method, instead, applies thermal energy from the periphery toward the center of the tumor. Therefore, in this case a minimum of two applicators are inserted within the periphery of the tumor in order to deliver the energy concentrically within the target. Among the three thermal ablation techniques reported herein, this strategy is implemented in clinical practice only by means of RFA delivering bipolar RF current. Centripetal convergent method provides safer tumor destruction, reliable safety margins, and the possibility to adjust the shape of ablated volume based on the tumor one and its location. Therefore, it is advisable for single tumors in risky locations or with a shape difficult to identify by means of imaging techniques. However, the centripetal strategy implies a greater procedural complexity due to the need of implantation of multiple probes with well-controlled directions and distances apart.

The following subsections are devoted to RFA, LA, and MWA description in terms of working principle and characteristics. Aiming to obtain an overall framework of these thermal ablative techniques and a comparison among them, the key aspects are also summarized in Table 1.

**Table 1:** Comparison among RFA, LA, and MWA

	Applicator	Working principle	Features	Advantages	Disadvantages
RFA	RF electrodes	Alternating electric current at high frequency (460÷500 kHz) induces orientation changes of the tissue ions, following the changes in the direction of the alternating current. Ionic agitation results in frictional heating.	**Influencing factors** RF settings (current density and application time), tissue type (electrical impedance). **Probes** Monopolar or bipolar, multi probes, hooked probes, internally cooled electrodes.	• Well-studied and clinically relevant ablation source • Impedance control • Reduced invasiveness with monopolar probes • Possibility of convergent centripetal approach with bipolar probes	• Moderate volume of necrosis • Temperatures not exceeding 100°C • Thermal conduction as first heating mechanism (charring dependence) • Need of an electrically conductive path
LA	Optical fibers	The interaction between laser light (operating in the NIR range) and tissue causes photothermal heating, dominated by absorption phenomenon.	**Influencing factors** Laser settings (λ, P, emission time and modalities), tissue type (thermo-optical properties). **Probes** ND:YAG (λ = 1064 nm) and diode (λ = 980 nm). Light is delivered by one or more quartz fibers (d = 300÷600 μm).	• Well suited for small tumors • Small dimensions of the applicator: compatibility with guiding endoscopes • All-dielectric applicators: compatibility with MRI.	• Difficult placement of the laser fibers • Limited light penetration in tissue and treatable tumors
MWA	MW antenna	Microwave radiations (typical frequencies are 915 and 2450 MHz) induce dielectric hysteresis, increasing the kinetic energy of the molecules and thus the temperature of the tissue.	**Influencing factors** MW settings (P, duration), antenna design, tissue type (water content). **Probes** Monopole, dipole, triaxial, choked, or slotted antennas. Cooling system usually included.	• High MW penetration in tissue • Large volumes of necrosis • No need of electric conduction (no limitations due to charring and wide range of treatable organs) • Synergistic capabilities by means of multiple antennas	• High bulk • High tendency of coaxial cables to heating • Need of cooling mechanisms along the antenna

### Radiofrequency ablation

The basic technique for RFA was described over a century ago by D’Arsonval, who, in 1891, first demonstrated that RF waves cause an increase of the tissue temperature.^19^ RF-based medical applications became widely popular since 1928, when Cushing and Bovie introduced the Bovie knife,^20^ consisting in a small knifelike electrode, a grounding pad, and an alternated current generator in the RF range. By varying the involved RF current, it could be used for cauterization or for cutting tissue. Therefore, the first generation Bovie knife represents a monopolar electrode like that used today for percutaneous RFA.

Although RF devices became already widespread, the electrophysiological principles of an RF-caused lesion were demonstrated only in 1976 by Organ, who explained for the first time the difference between electrocautery and RF technique.^21^ Unlike electrocautery, for which a probe is heated by passing current through a heater element in its tip and the latter is applied to the tissue, with the RF technique the primary source of heat is the tissue itself rather than the probe. More specifically, a high-frequency alternating electric current (460÷500 kHz) applied to the tissue, incapable of inducing molecular rotation,^22^ flows from the uninsulated portion of an electrode finding the path to the grounding pad and activates a thermal heating by Joule effect depending on the tissue electric impedance, as well as the current density and application time. The ions in the tissue surrounding the electrodes attempt following the changes in the direction of the alternating current, and this ionic agitation results in frictional heating.

In the clinical arena, RFA has been used for treating a variety of neoplasms, including osteoid osteoma, hepatocellular carcinoma (HCC), renal cell carcinoma, hyperfunctioning parathyroid adenoma, and hepatic, cerebral, and retroperitoneal metastases from a variety of primary tumors.^23^^,^^24^ RFA can be performed with either monopolar or bipolar techniques, where monopolar ones are more commonly used for tumor ablation. When a monopolar technique is used, a single interstitial electrode (or a group of electrodes) is used to deliver current at the tumor site, while a large dispersive electrode (i.e., grounding pad) on the skin, usually placed on the patient’s thigh, completes the electrical circuit through the body. When a bipolar technique is used a second (passive or ground) electrode is placed within 5 cm of the active electrode. Bipolar mode generally has the advantages of: i) a focused and more effective heating in the area between the electrodes, ii) reduced dependence on background conductivity, iii) no need for grounding pads, and iv) capability of convergent centripetal ablation, as previously mentioned. However, bipolar mode needs additional electrode insertions, becoming more invasive, and often saline infusion to improve results. On the other hand, monopolar mode has the advantages of i) a wider zone of heating around each electrode; ii) limited invasiveness; and iii) wide clinical availability.^25^

**Figure 1:**
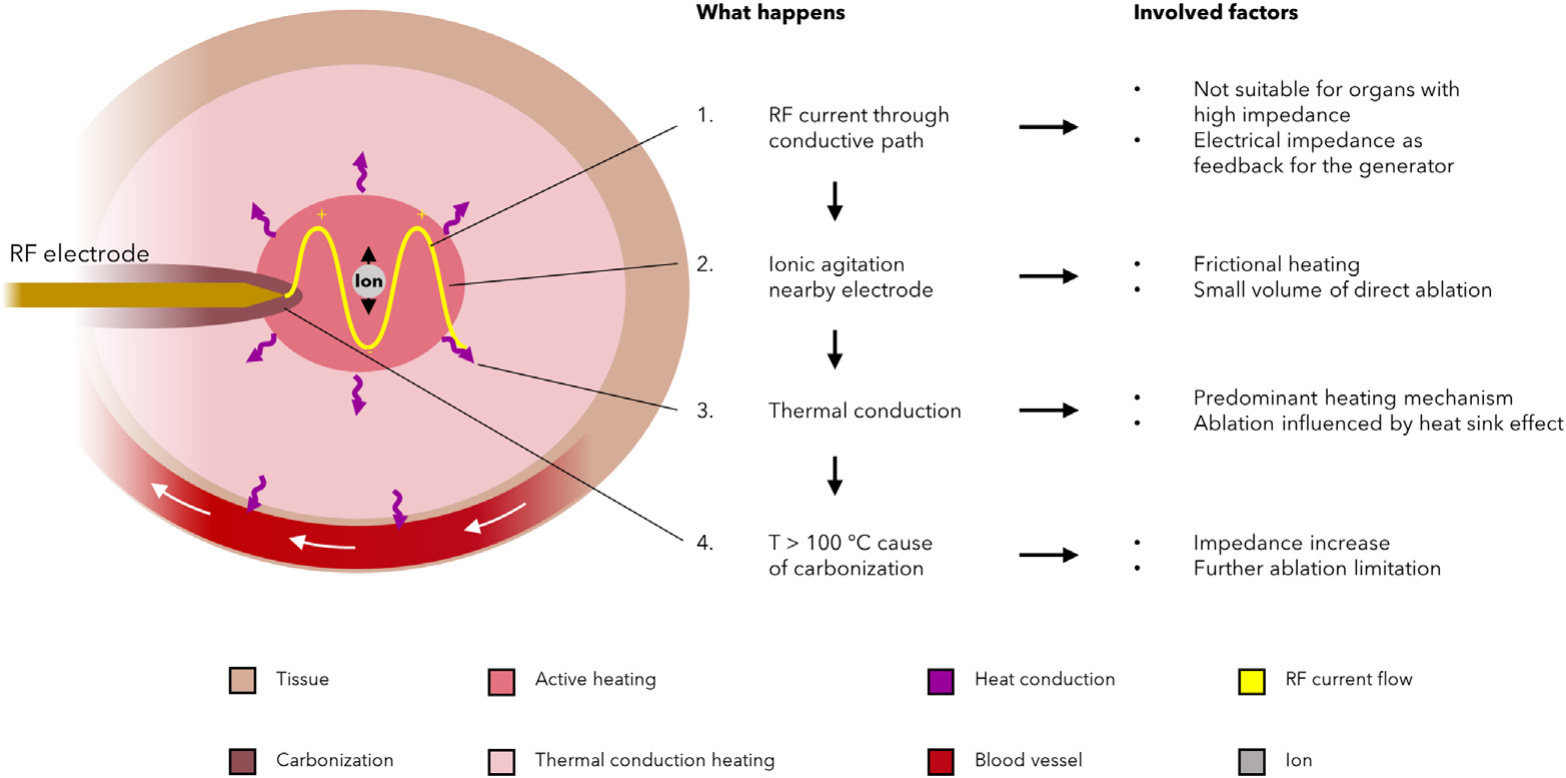
Mechanisms of RFA Schematic of the heating mechanisms and their implications caused by RFA inside the tissue surrounding the RF electrode.

Apart from the kind of involved technique, in general RFA effectiveness is limited by several characteristics, which are intrinsic in its working principles, as synthesized in Table 1 and schematically described by Figure 1, showing the heating mechanisms involved by RFA nearby the applicator and their implications. First, ionic agitation, and thus tissue heating, is greatest in the areas of the highest current density, i.e., closest to the active electrode tip, so that necrosis is limited to a relatively small volume of tissue surrounding the RF electrode,^23^ which is represented as the active heating zone of Figure 1. Moreover, the RF current application leads to the coagulation phenomenon as well as to the local tissue charring, which in turn increases the tissue impedance. Since RFA needs an electrically conductive path to carry out RFA,^25^ high electrical conductivities (i.e., low impedances) allow more current flow and more power to be applied from the generator, while low electrical conductivities (i.e., high impedances) inhibit current flow. Therefore, charring phenomenon, by increasing tissue impedance, inhibits the flow of RF current and further ionic agitation, and thus limits the extent of tissue injury by creating a heat trap. This phenomenon is explained by considering that the predominant heating mechanism involved by RFA is thermal conduction rather than direct heating, which corresponds only to the portion immediately around the electrodes. Heating caused by thermal conduction, indeed, affects a larger portion of tissue with respect to direct (active) in the schematic of Figure 1. Therefore, in the presence of charring, the resultant eschar forms a highly effective insulator around the electrode and conduction effect is dramatically reduced, causing small volumes of ablation. Under this condition, RF generators can implement a “safe mode,” causing the power supply discontinuities and the interruption of the ablation procedure.^26^

It is worth noting that charring dependence not only impacts on RFA effectiveness, but potentially increases tissue interstitial pressures. In addition, since carbonization happens when the temperature exceeds 100°C, it also limits the temperature gradient achievable within the lesion.

Aiming to expand the thermal lesion size, several approaches have employed, including: i) the use of multiprobe, hooked, and bipolar needle arrays, ii) intraparenchymal injection of saline before and/or during RF application, iii) internally cooled RF electrodes, and iv) algorithms for RF current application that maximize energy deposition but avoiding tissue boiling, charring, or cavitation.^23^^,^^27^ In particular, with the internally cooled electrode, 14÷18-gauge thicker, chilled perfusate is circulated through the internal lumen of the probe during RF application. By cooling the electrode tip during RFA, it is possible to increase generator output and, at the same time, to prevent tissue boiling and cavitation immediately adjacent to the needle. In this way, the extent of coagulation can range from 1.8 cm to 3.6 cm with single internally cooled electrodes and from 4.5 cm to 7.0 cm by using electrode clusters.^28^

Furthermore, given that most of the heating zone is ascribed to thermal conduction phenomenon, RFA results more limited by the so-called heat sink effect caused by the blood flow through the blood vessels near to the ablation target, as described in further detail in Paragraph 3.2. This limitation becomes particularly significant in highly vascularized organs, such as liver.

The RFA need of an electrically conductive path represents a drawback also because it limits the range of treatable tumors. Indeed, RFA is not suitable for organs having low electrical conductivity (i.e., high impedance) and poor thermal conduction, such as bones and lungs.^29^ However, at the same time, being impedance-dependent, RFA is the only ablation technique among the ones addressed in this paper which can be robustly modulated by a continuous impedance measurement. The latter, indeed, is used and displayed as feedback signal for most of commercially available RF generators: as temperature reaches 100°C and boiling occurs, the impedance increasing read by the generator limits further deposition of electricity into the tissue.

### Laser ablation

The first clinical use of lasers for the treatment of malignancies was reported more than 50 years ago.^30^^,^^31^^,^^32^ After ten years, the first coagulation zone was obtained in a patient with lung carcinoma, representing the first example of laser energy use as ablation technique.^33^ Although LA is a recent thermal therapy, it already represents the selected technique for a broad range of carcinomas, such as those in the liver, pancreas, kidneys and thyroid, to name the most relevant.^34^

Because laser light is coherent and monochromatic, it can be highly collimated or focused with little energy loss, enabling large energy densities to be transmitted in narrow beams over long distances without significant losses. From the medical purposes, the extremely collimated behavior and the high degree of monochromaticity are crucial properties, implying the possibility of precisely ablating soft tissue and involving selective interactions with tissues.^35^ When laser light strikes the tissue, the main result is thermal effect. More specifically, since biological tissue is a turbid media, absorption, scattering, reflection, and transmission occur, where the former produces biological effects, whereas the other phenomena determine where the light goes. Moreover, scattering increases the mean path length of the photons within short distances, and thus the probability of local absorption. At the same time, scattering limits the depth of penetration because it can occur backward as well as forward.^36^ Because of the weak wavelength dependence for scattering in the near infrared (NIR) region of the spectrum, absorption of the photons in laser light dominates the photothermal heating of tissue, depending on the laser wavelength.^37^ Due to light absorption, temperatures up to 150°C are reached, leading to coagulative necrosis. The probability per unit distance of absorption is referred to as the absorption coefficient, $\mu_{a}$, and the probability of scattering per unit distance is the scattering coefficient, $\mu_{s}$, being $\mu_{s}$ much larger than $\mu_{a}$ in case of turbid media. For human tissues, the values of $\mu_{a}$ and $\mu_{s}$ coefficients lie in the ranges 0.001÷10 mm^−1^ and 1÷100 mm^−1^, respectively. For application to photon transport, the anisotropy g of the scattering must be considered, so that transport effective scattering coefficient is ${\mu_{s}}^{\prime }=\mu_{s}\left(1-g\right)$. During LA, ${\mu_{s}}^{\prime }$ has been noted to increase substantially at high temperatures for many tissues, increasing the local heat generation. Therefore, besides the laser settings (i.e., wavelength, power, duration of irradiance, emission modalities of the applicator, pulse duration and repetition frequency when pulsed emission is used), the temperature profile in the irradiated tumorous tissue and the overall biological effects of laser energy also depend on the optical properties of the involved tissue (i.e., $\mu_{a}$, $\mu_{s}$, g). Moreover, both anatomical and thermal characteristics, such as the presence of blood vessels and perfusion or local tissue composition, and its heat losses due to conduction and convection, play a crucial role in light-tissue interactions.^31^^,^^38^

Neodymium:Yttrium Aluminum Garnet (Nd:YAG, wavelength of 1064 nm) and diode (wavelength in the range of 800÷980 nm, typically 980 nm) lasers are the most commonly used, as the penetration of light is optimal in the NIR spectrum showing a good balance between absorption and penetration into the tissue (in the order of millimeters).^39^ On the contrary, for superficial treatments, CO_2_ lasers, thulium lasers or holmium lasers (Ho:YAG) with wavelengths of 10600, 2016 and 2100 nm, respectively, are preferred due to their lower penetration depth (from 0.1 mm to 1 mm approximately).^35^ Light is delivered through flexible quartz fibers with a diameter from 200 to 600 μm.^35^^,^^40^ A bare-tip fiber provides an almost spherical thermal lesion of 12÷15 mm in diameter, and a beam-splitting device or a multi-source device allows the use of multiple fibers, simultaneously delivering the light into each single fiber. Bare tip fibers are usually inserted through 21-gauge needles into the lesions. Typically, one or two fibers are used to treat nodules up to 1.5 cm in diameter, three fibers to treat nodules from 1.5 to 2.5 cm, and four fibers with tips arranged in a square configuration to treat nodules with a diameter greater than 2.5 cm.^7^ In addition, to treat larger nodules the pullback technique can be used.^41^

**Figure 2:**
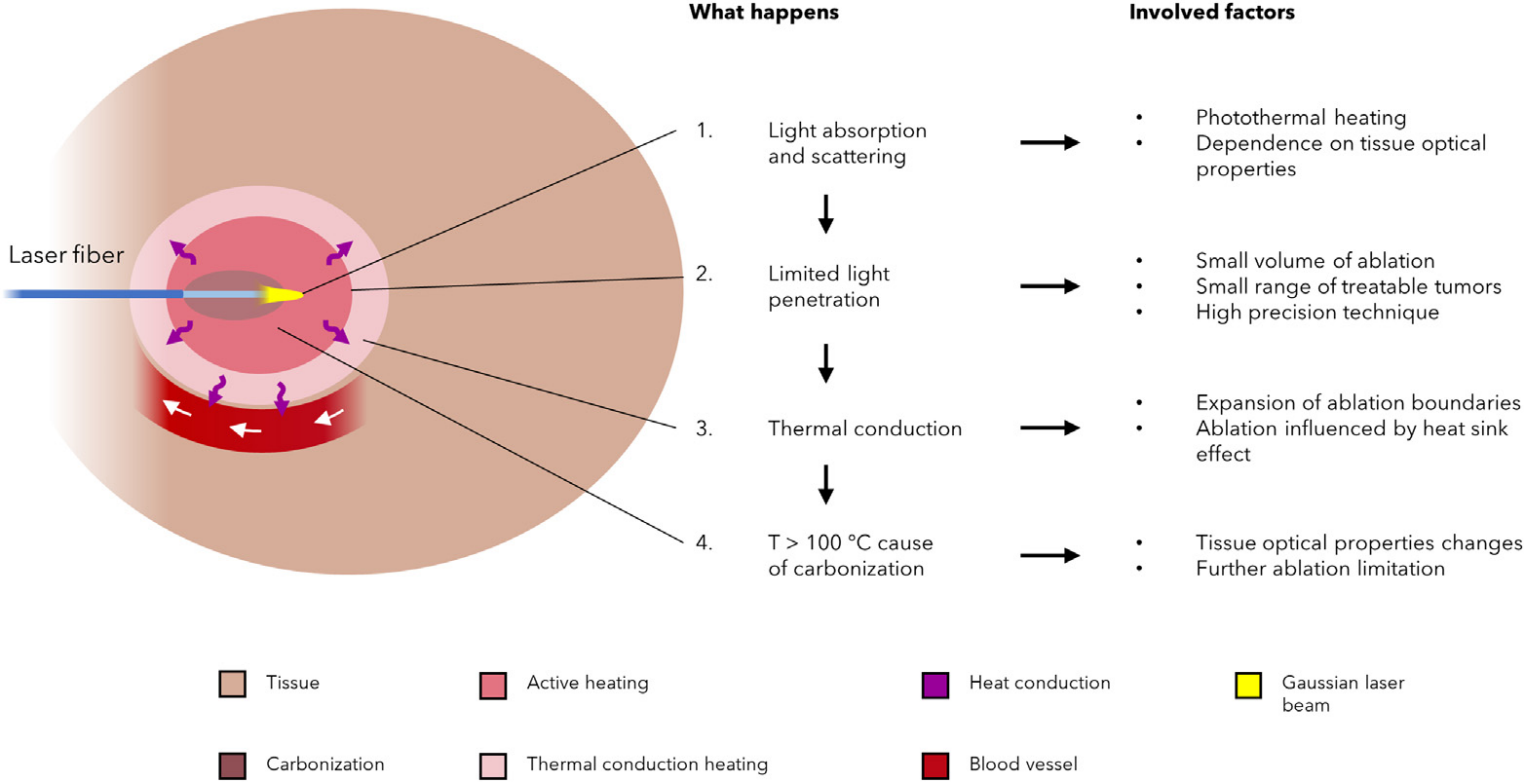
Mechanisms of LA Schematic of the heating mechanisms and their implications caused by LA inside the tissue surrounding the laser fiber.

Features, advantages, and drawbacks introduced by LA are outlined in Table 1, whereas Figure 2 schematizes the ablation process and the involved factors. The dependence of the laser-tissue interactions on thermal characteristics of the involved tissue results in the fact that laser light damages the tissue due to its absorption of light as well as through heat conduction that follows the energy absorption into the tissue. If the delivered energy is high enough, indeed, the heat conduction contributes to the lesion progress since it can induce the exceeding of tissue temperature threshold for permanent damage.^42^ Such dependance on heat conduction, if on the one hand helps to achieve a larger ablation volume, can represent a drawback insofar as factors such as convective phenomena due to blood perfusion influence the thermal gradient inside the lesion by the heat sink effect.^7^

Furthermore, the correct placement of the laser fibers used as applicators can result technically difficult, particularly if more than two fibers are needed, and should be performed by very skilled operators. However, as highlighted in Figure 2, the greatest issue of LA lies in the limited penetration of light in most tissues, which is significantly less than that of radiofrequencies or microwaves. This constraint rules out the use of LA for tumors of thickness more than about 10 mm.^31^ Indeed, an optimal temperature distribution requires that the optical penetration depth is of the same magnitude as the tumor thickness. Furthermore, high laser powers can cause water vaporization and thus tissue carbonization nearby the laser applicator, and this phenomenon further reduces the ablation size. On this matter, it is worth noting that coagulation and carbonization change the optical properties of tissue in terms of absorption and scattering coefficients depending on the tissue type, as reported in Figure 2. For these reasons, the cooling of the laser fiber by means of the dual-lumen design can be implemented to circulate the cooling liquid (often a physiological solution at room temperature) around the applicator.

On the other hand, the typical LA disadvantage, i.e., the limited penetration of laser light inside the tissue and its consequences, constitutes at the same time the strong point of this technique and the reason why it is the preferred one when treating small and well localized tumors (e.g., brain or ocular tumors). Indeed, the small size of the optical fibers serving as laser energy applicators enables to place them directly into the target organ with high precision. Moreover, the fibers can also easily be guided through the biopsy channel of standard endoscopes, and their distal end can be inserted directly into tumors of internal hollow organs such as kidneys. Finally, LA shows the great benefit for which it is the only ablation approach fully compatible with magnetic resonance imaging (MRI) by making use of all-dielectric applicators (i.e., optical fibers).

### Microwave ablation

Microwave ablation (MWA) is one of the most recent developments in the field of tumor ablation. This technique allows for flexible approaches to treatment, including per-cutaneous, laparoscopic, and open surgical access [22]. The initial studies of microwave coagulation were developed at the beginning of 1980s to achieve hemostasis along the plane of transection during hepatic resections [42]. Then, the first clinical application of microwaves for tumor ablation were described in the early 1990s in [43,44], in both cases for patients affected by HCC. Although not well-studied such as RFA technology, now MWA has expanded its clinical use for many kinds of cancer, from small HCC to large liver cancers greater than 5 cm, and for several organs including kidney and adrenal organs, spleen, thyroid, lung, breast, abdominal wall, and uterus [45].

MWA uses electromagnetic waves and dielectric hysteresis to generate heat inside the tissue. The involved electromagnetic fields are typically in the range of 900÷2500 MHz. More specifically, current MWA technology employs electromagnetic wave frequencies of 915 MHz or 2450 MHz, as allowed by the Federal Communications Commission [46]. The basic MWA system consists of three components, i.e., MW generator, power distribution system and antennas. Power is generated with a magnetron device or solid-state sources, and the electromagnetic field is emitted through the exposed portion of the antenna. Distribution of electromagnetic energy from the generator to the antenna is mostly accomplished through a coaxial transmission line, which has excellent propagation characteristics. MW field then radiates from the emitting point of the antenna through the surrounding tissue and, due to the phenomenon of dielectric hysteresis, the field forces the polar molecules with intrinsic dipoles (predominantly water) within the tissue to continuously realign with the oscillating electric field. The rotating dipoles increase the kinetic energy of the molecules, increasing in turn the temperature inside the tissue [17]. As a result of an MW radiation hitting, indeed, the electric charge of the polar molecule flips back and forth between 2 and 5 billion times per second, depending on the frequency of the employed MW system. These spinning molecules interact with neighboring tissues, transferring a portion of their kinetic energy [22]. As a result, there is conversion of rotational energy into heat.

**Figure 3:**
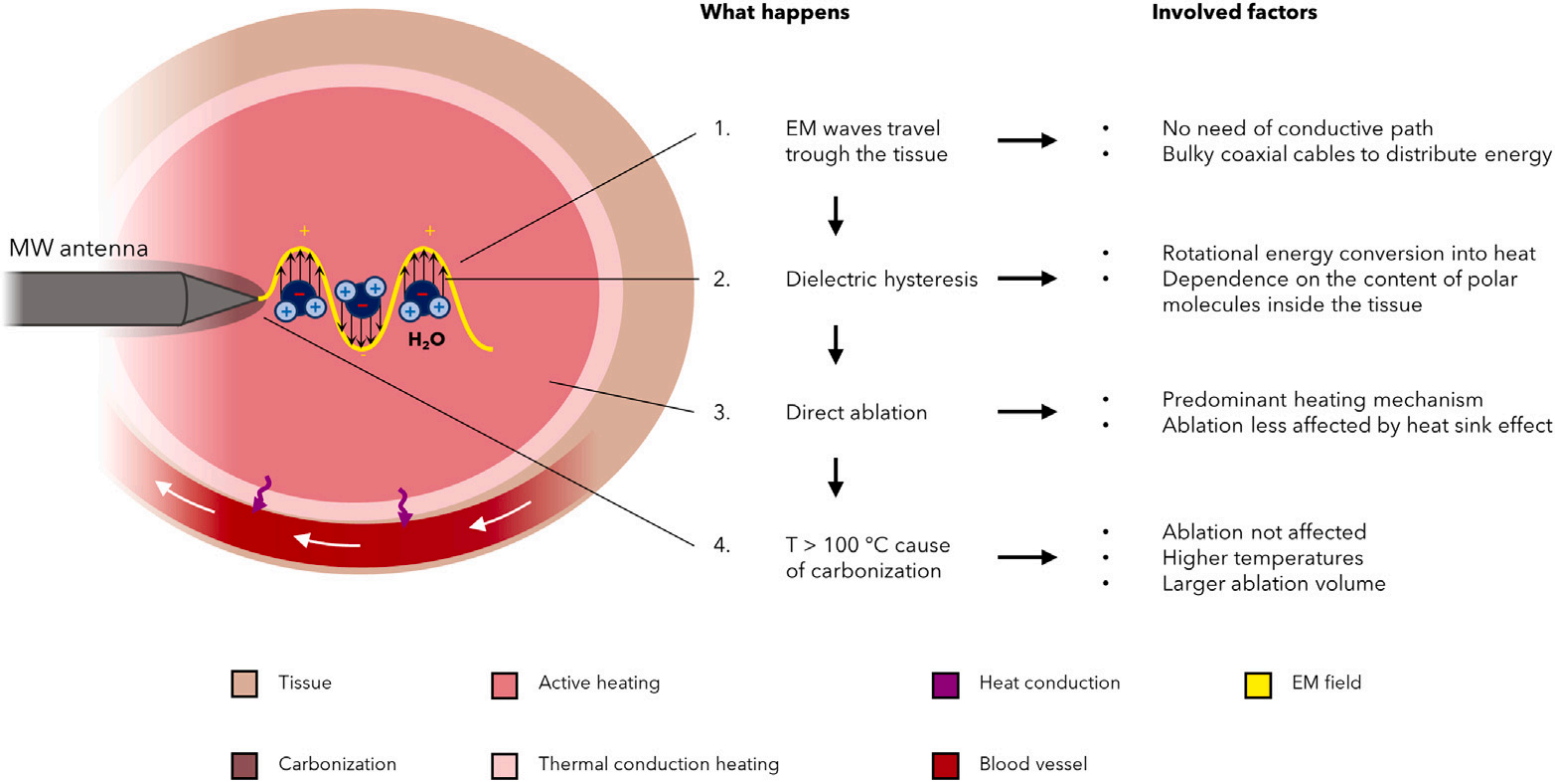
Mechanisms of MWA Schematic of the heating mechanisms and their implications caused by MWA inside the tissue surrounding the MW antenna.

Table 1 includes the main characteristics of MW, allowing for a direct comparison with the previously described techniques of thermal ablation, whereas Figure 3 reports a schematic of the MW ablation process.

Size and shape of the ablation zone generated by any MW antenna inside the surrounding tissue depends on the antenna design, and, as for the other previously described thermal sources, tissue type (considering the changes in its properties during the ablation), thermal conduction from the active heating zone, and thermal sinks (i.e., heat sink) caused by nearby structures such as blood vessels, as schematically represented in Figure 3. In particular, to optimize the performances, antenna design is a balance among power efficiency, tissue heating pattern, and diameter, a tradeoff to produce the desired result, in terms of sufficiently extended volumes of ablation and minimal invasiveness of the procedure.^29^ Common designs for MW antennas include monopole, dipole, triaxial, choked, or slotted antennas, but research has been mainly focused on coaxial-based interstitial antennas.^43^ Due to the coaxial cable construction, antennas with a small diameter can hardly handle higher power levels without unwanted thermal damage to tissues around the proximal antenna shaft. Therefore, cooling jackets and antenna shaft cooling systems have been spreading in the current MWA commercial systems in order to reduce such heating, eliminate skin burns, increase the possibility to use smaller-diameter antennas (i.e., less invasive applicators), deliver higher powers for longer times, and, in turn, produce larger ablation zones. Circulation of chilled saline solution or distilled water at 0°C by a peristaltic pump is the most used method for cooling the antenna shaft, but compressed gas is also used since the rapid decompression of carbon dioxide gas causes the Joule-Thomson phenomenon to occur at the probe tip, with gas venting up the shaft. The high cooling capacity of this system allows the use of high-power generators (140 W) while maintaining small shaft diameters (17-gauge).

MWA has shown great promise for tumor treatment exhibiting many benefits over other thermal ablation techniques. One potential advantage of MWA over RFA and LA is that investigational *ex vivo* studies have shown greater tissue penetration and larger zones of coagulation-necrosis. This finding is most pronounced with MW antenna integrated with a cooling system. Indeed, in contrast to RFA, MWA does not rely on electric currents and conduction through tissue, and so they radiate through all biological tissues, including those with high electrical impedance. Therefore, temperatures above 100°C are usually administered without the concern about the limitations to complete target heating due to tissue desiccation and charring. Moreover, MWA does not need grounding pads or other auxiliary components, and it is less affected not only by tissue carbonization, but also by heat sink effect caused by the near blood vessels. Indeed, this ablation technique is mainly based on direct heating, as represented in Figure 3, and thus less dependent on thermal conduction and blood perfusion, becoming more advantageous for treating tumors of highly vascularized organs such as liver. In fact, at certain frequencies MWA can induce active heating inside the tissue up to 2 cm away from the antenna.^17^ A further advantage of MWA is the ability to induce a synergistic increase in lesion size, rather than additive when multiple antennas are applied, amplifying the ablative effect. Indeed, phasing the electromagnetic waves constructively, the heat generated is proportional to the square of the number of antennas. This synergistic capability is not available with RFA, since multipolar RF fields would need to be continuously switched between pairs of monopolar electrodes.^29^

On the other hand, it is worth noting that MWA systems are more cumbersome than RFA and use larger coaxial cables, likewise more bulky and more prone to heating than the simple wires used in RFA. Large cable diameters cannot be avoided because as cable diameter and surface area decrease, more power loss and increased cable heating happen. This leads to the need of a cooling mechanism to protect the superficial structures along the antenna. Cooling systems integrated in the antenna also prevent skin burning at the point of antenna entry caused by the heating of the antenna shaft due to the power reflection along itself. Moreover, at present MWA as well as LA cannot be monitored by any tissue feedback, unlike RFA monitoring by continuous tissue impedance measurements.

### Choosing among radiofrequency ablation, laser ablation, and microwave ablation

RFA, LA, and MWA all require insertion and positioning of applicators (or probes) to deliver the thermal energy *in situ*. Some techniques require multiple applicators to be inserted (e.g., for multi-bipolar radiofrequency and in case of a plurality of laser fibers for treating multiple nodules), whereas a single applicator is often sufficient for others (such as monopolar RFA and MWA). These methods are conceptually very similar but are distinguished from each other in practice through the technologies they use, and there is no universally effective ablation technique in interventional radiology. Indeed, the advantages of a given technology are often consubstantial with its limitations, and each method can offer the best benefit/risk balance in a specific clinical situation.^18^

Therefore, each of these technologies has its own strengths and weaknesses when applied to the localized treatment of tumors in a variety of solid organs. Anyway, to determine the optimal source of thermal ablation in a specific circumstance, it is worth considering some decisional element useful to guide such choose. For most start-up tumor ablation practices with limited available capital to invest in a device, RF technology may be the most versatile modality upon which to begin building a practice. As described in the previous paragraphs and summarized in Table 1, MW technology can generate larger zones of tissue destruction with shorter treatment times, and it is less susceptible to variation in the morphology of the treatment zone due to heat-sink effects from adjacent vasculature. However, with larger zones of tissue destruction comes the increased risk of potential complication owing to collateral injury to adjacent non-target organs. Therefore, the use of this technology may be more suited for practices whose operators have a considerable amount of experience performing thermal ablations, often using percutaneous image-guided techniques.

Although RFA represents the “historical” and more experienced thermal ablation technique, both MWA and LA have been demonstrated to be as effective and safe as RFA when performed by skilled operators.^7^ In most centers of interventional oncology or radiology, the choice among the available techniques usually depends on the physicians’ preference and experience. When all the three techniques are viable in terms of clinical resources, the selection of the most suitable one depends on the patient’s health conditions, his/her characteristics and comorbidities (e.g., the presence of a pacemaker is a relative contraindication to RFA), on the electrical, optical, and thermal properties of the tissue to treat, and on tumor features such as size, location, and proximity to critical anatomical structures such as vital organs, blood vessels, air or bile ducts. Generally, LA is suitable in case of tumors whose dimensions do not exceed 1.5 cm, RFA is appropriate for the treatment of lesions within the liver and kidney that are less than 2 cm in diameter, whereas MWA is applicable to a broader spectrum of tissues, including lung, liver, kidney, and bone.^44^

**Figure 4:**
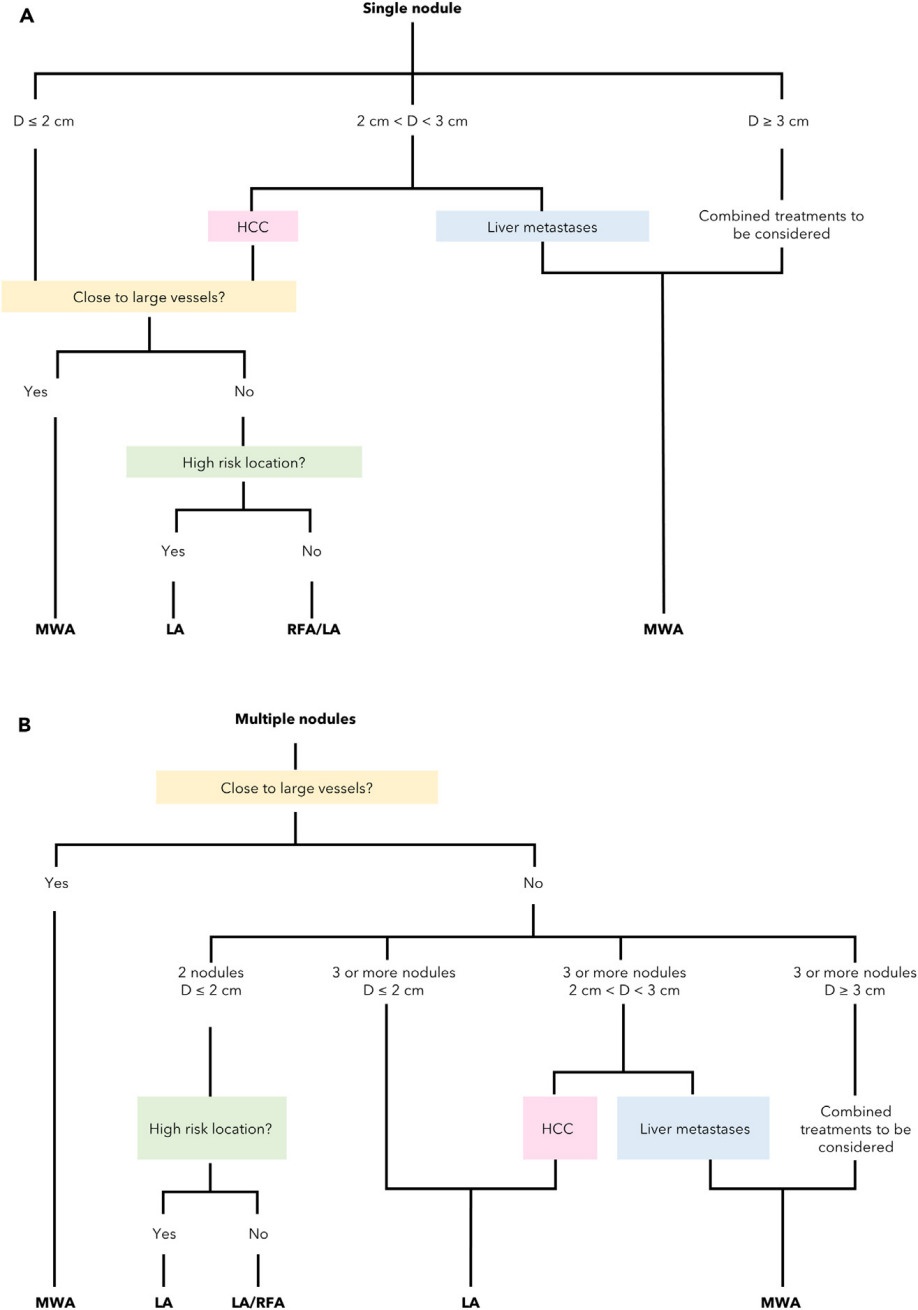
First example of ablation technique decisional algorithm Diagram of an example of decisional algorithm^7^ for the choice of the appropriate thermal ablation technique in case of HCC carcinoma and liver metastases for. (A) single nodule. (B) multiple nodules.

In case of liver tumors, Tombesi et al.^7^ suggested an algorithm, reported in Figures 4A and 4B, to tailor the choice of the thermal ablation technique on the single patient aiming to achieve the best clinical outcome. According to such algorithm, both RFA and LA are cheaper than MWA and should be preferred unless the tumor has a large diameter or it is close to large vessels, since MWA is capable of larger zones of coagulation-necrosis and less affected by the heat-sink effect in the presence of blood vessels with respect to the other techniques.

As can be inferred from the diagram, LA is suggested when the nodule has small dimensions and lies in a difficult or risky anatomical position, as the needles used to insert the fibers are considerably finer than RFA electrodes or MW antennas. In case of HCC with a diameter (i.e., denoted by D in Figure 4) ranging from 2 cm to 3 cm, multiple overlapping insertions can be needed for RFA, and at least three fibers should be used for LA. Multiple small lesions with a maximum diameter of 2.5÷3 cm, should be preferably treated with LA, as the ablation area can be diversified according to the lesion size using from one to four fibers, thus sparing the healthy tissue as far as possible. Finally, also for multiples nodules, MWA should be preferred when the tumors are close to large vessels or have a diameter greater than 3 cm. However, one should consider that this algorithm only applies to liver tumors. Moreover, it does not consider the specific patient to be treated.

Other decisional algorithms have been developed based on the organ to treat, therefore considering not only liver tumors. In this regard, Hinshaw et al. proposed a set of flowcharts recommending the most suitable technique among RFA, MWA, and cryoablation for liver, kidney, lung, and musculoskeletal system.^45^

**Figure 5:**
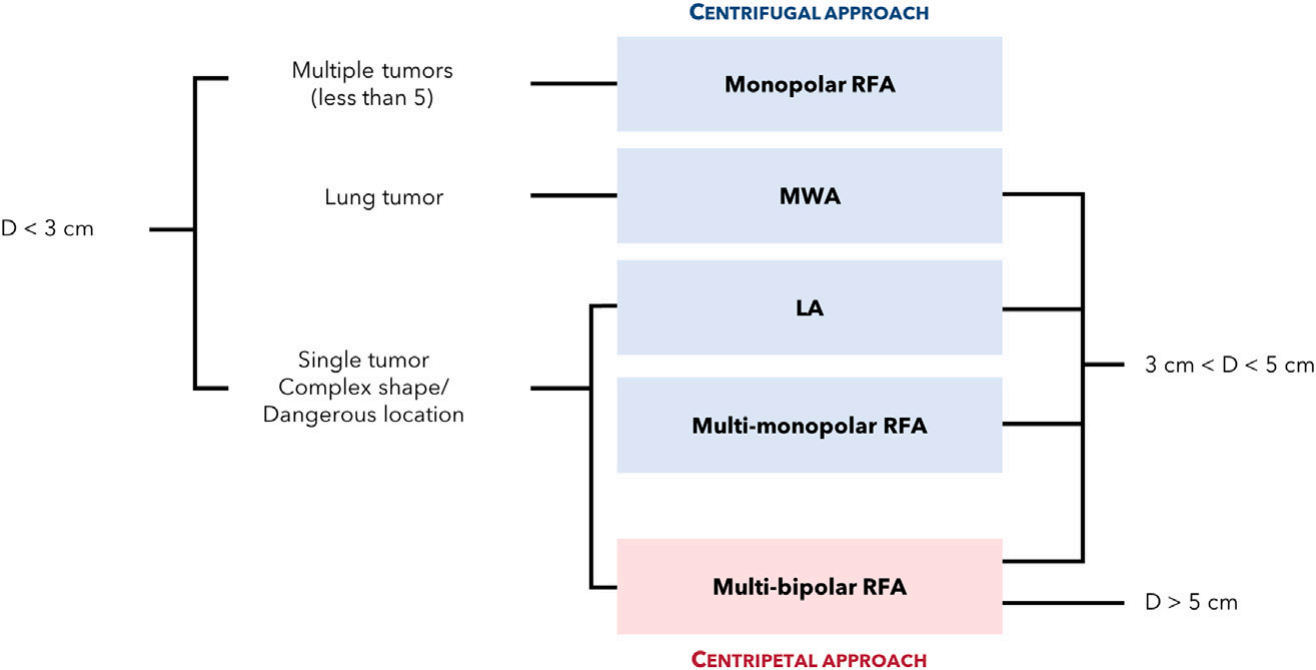
Second example of ablation technique decisional algorithm Diagram of an example of decisional algorithm^18^ for the choice of the appropriate approach of thermal ablation based on tumor features.

The choice among the available thermal ablation techniques can address not only the type of energy source, but also the kind of approach, e.g., whether to opt for a centrifugal radial technique or a centripetal convergent one, as described by Seror in.^18^ In such article, Seror proposes a rationale to choose the most appropriate thermal ablation technique based on the tumor features, firstly its size. Larger tumors with respect to the ones considered in the decisional algorithm proposed in,^7^ i.e., with diameters above 3 cm, are considered, as reported in Figure 5, which describes such rationale.

## Temperature monitoring need

Although efficacy, functional outcomes, and improvements in mortality over conventional treatments vary substantially depending on the modality and among different tumor types, all the available techniques of thermal ablation have to face some common issues.^17^ The typical risks introduced by thermal ablation are the incomplete tumor destruction, the unintended thermal damage to nearby healthy structures, and the recurrence phenomenon. In essence, the key problem shared by all the available techniques lies in their lack of treatment selectivity. Such intrinsic issue, indeed, leads to the main severities previously mentioned.

During the last decades, several techniques have been investigated and exploited to overcome this obstacle to achieve a safe and complete tumor thermal ablation. As a matter of fact, regarding the safety of the procedure, several reports in the literature have described significant collateral thermal damages during ablation, such as bowel perforation during the treatment of liver, kidney, and prostate tumors, or severe cartilage damages during the thermal ablation of musculoskeletal tumors near articulations.^46^ Therefore, to spare the healthy tissues which surround the tumor, different thermo-protective measures have been applied, such as percutaneous insulation techniques such as gas dissection, balloon interposition, hydrodissection, endoluminal warming/cooling, and organ filling/emptying.^46^^,^^47^^,^^48^

Despite thermal and physical insulation strategies can represent valid and relatively easy procedures to protect the healthy anatomical structures surrounding the tumor, these techniques introduce several drawbacks, e.g., gas dissection is not suitable for ultrasound-guided interventions because the gas causes a ring-down artifact and obscures the image, fluid injection is not suitable for cryoablation because it can freeze upon contact with the ice ball and thus increase the risk of thermal damage, and balloon interposition is mainly used for bowel displacement whereas it should not be used for the protection of the neural structures during the ablation of spinal lesions since it can be traumatic for the neural structures. Moreover, these strategies may not be enough to guarantee the thermal ablation safety and, above all, the control of the procedure. The risk of an incomplete ablation, indeed, remains, potentially leading to tumor recurrence. Hence the need of temperature monitoring during the entire treatment. An accurate measurement of tissue temperature can be particularly beneficial to improve treatment outcomes because it can be used as a clear endpoint to achieve the necessary temperature within the tumoral mass and at the boundaries of the ablation zone for a complete tumor ablation and minimized recurrence. For these reasons, although the lack of selectivity in tumor ablation treatments still remains a hurdle to overcome unless specific kinds of nanoparticles are employed and accumulated into the cancerous tissues, as reported in the following subsection, temperature monitoring during thermo-ablative treatments can help in addressing the lack of selectivity which is intrinsic of thermal ablation by providing information about the current heating of the target volume of tissue.

### Role of temperature monitoring

**Figure 6:**
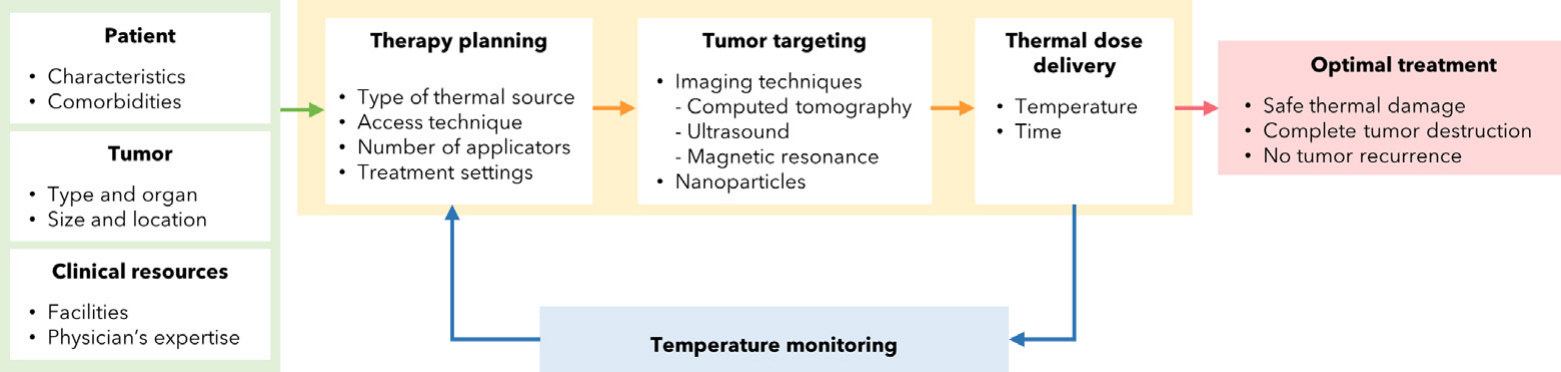
Thermal ablation process Diagram of the thermal ablation process describing its typical steps, from therapy planning to tumor targeting and destruction, including the feedback derived from temperature monitoring to control the procedure.

The role of temperature monitoring during thermal ablation treatments is represented by the feedback loop in the process schematically described in Figure 6 and discussed in the following.

The green block in Figure 6 embeds the preliminary steps to implement any thermal ablation technique. Therefore, this stage considers the determining factors (discussed in Section 2.4) that lead to the appropriate therapy planning: i) patient-dependent features, i.e., her/his general health conditions, comorbidities, ii) tumor characteristics, i.e., its type, size, and proximity to critical anatomical structures, and iii) clinical resources such as the available facilities and the physician’s expertise and preferences.

Thermal ablation implementation corresponds to the yellow block in Figure 6, and its first stage is represented by therapy planning, which concerns not only the choice of the most suitable type of treatment, but also the applicator selection (e.g., among monopolar, bipolar, or multi-tine probe for RFA, cooled or not cooled antenna for MWA, and single or multiple laser probes for LA). Moreover, access technique (e.g., laparoscopic, endoscopic, percutaneous, or extracorporeal), number of applicators, which in turn depends on tumor dimensions, and treatment settings (e.g., power and time duration) are decided in this phase.

Then, tumor targeting is needed to visualize and localize the target region. This step allows to appropriately insert the applicator(s) into the target and apply the thermal energy within it. In the clinical practice, indeed, thermal ablation can be performed through imaging guidance such as ultrasounds (US), computed tomography (CT), or magnetic resonance imaging (MRI). Ideal attributes of a targeting technique by using imaging guidance include a clear delineation of the tumor(s) and the surrounding anatomy, coupled with real-time imaging and multiplanar interactive capabilities. For example, US and some MRI systems have all of these features.^49^ Another tumor targeting strategy resorts to nanomedicine, namely the application of nanomaterials (i.e., nanoparticles, NPs) with a size range of 1÷100 nm, for diagnosis, monitoring, and treatment of diseases. With the advent of “theranostic” concept, indeed, NPs with both diagnostic and therapeutic properties are emerging. Therefore, NPs have been widely employed in anticancer therapy, in particular for the delivery of cargo molecules (i.e., imaging agents, genes or chemotherapy drugs), or alone, for example for target purposes.^50^ The main reason why NPs are such attractive for cancer targeting lies in their capability to spontaneously accumulate into cancerous tissues thanks to the enhanced permeability and retention effect. Tumor tissue, indeed, is highly vascular, and the ill-formed blood vessels are highly permeable to different macromolecules such as lipids, plasma proteins, and NPs. Moreover, the poor lymphatic clearance of tumors causes the increased retention of these compounds within them.^51^ Once accumulated into the tumor, NPs can generate and specifically enhance the heating capacity at the tumor region. Due to their magnetic and optical properties, indeed, NPs can trigger heat increase in tumor regions by absorbing near-infrared light (NIR), electromagnetic, or radiofrequency waves. Moreover, surface-functionalized nanomaterials can specifically bind to the cancer cell and allow selective heating and destruction of the tumor.^52^ In this way, NPs allow thermal ablation selectivity, but not only: they facilitate heat propagation toward the outer borders of the tumor, maximizing the volume of cell death by enhancing rate and radial extent of heating, thereby ensuring a complete ablation. Furthermore, NPs usually allow faster ablation times with lower input power, while the surrounding healthy tissue stays at a safe temperature.^53^^,^^54^

After therapy planning and tumor targeting, thermal energy is released, and a certain thermal dose is delivered to the tissue by the thermal source through the applicator. This is the last step of thermal ablation implementation and represents the accomplishment of the minimally invasive intervention. In the 1987, Gerner^55^ suggested and discussed two physical definitions of thermal dose for heating therapies of tumors: i) a fundamental definition, based on thermodynamic considerations, which describes thermal dose as a driving force for the reactions occurring in any system at a given temperature, function of the basic properties of the medium and temperature, and ii) a currently more practical definition for the clinical applications of hyperthermia in cancer therapy, which simply lies in a given temperature for some time. The delivered thermal dose represents the thermal ablation outcome, being the responsible of cell necrosis and tumor destruction. Thermal dose concept emphasizes the importance of controlling time-dependent temperature distributions within the target tissue^55^ and the meaning of the following step of thermal ablation process, i.e., temperature monitoring, as discussed hereafter.

Tumor localization and targeting, while accurate, alone are not sufficient to ensure a safe and complete thermal dose delivery. Hence the need for a feedback loop to the process in order to realize if the ablation outcome is as expected and, otherwise, to adjust the delivered energy settings during the treatment. In other words, the feedback loop allows the procedure control based on the driving parameter of thermo-ablative techniques, i.e., temperature distribution inside the lesion. In Figure 6, indeed, feedback is provided by temperature monitoring (blue block) during the entire procedure, since in this way thermal dose, i.e., temperature as the function of time, can be measured and adjusted by acting on the therapy planning block of the diagram to obtain the desired temperature distribution. Temperature feedback results in an optimal treatment in terms of completeness of ablation, delivering a safe and accurate thermal dose (and thus thermal damage), and avoiding tumor recurrence. As the increase of temperature is typically quick in thermal ablation changing with time and space, a rapid refresh rate with high spatial and moderate temperature resolution (1÷2°C) is required over a large dynamic range.^56^ In particular, good time resolution is essential in thermal ablation techniques where tissue temperature increases fast (e.g., during RFA). Moreover, the measurement range must cover temperatures higher than 100÷120°C and distributed or three-dimensional (3D) measurements with high spatial resolution (e.g., 1÷2 mm) extremely contribute to achieving a full description of the temperature gradient within the organ.^57^ Furthermore, regarding spatial resolution, the temperature gradient around the applicator represents a greatly relevant factor to be considered: the sharper the thermal gradient, the better the spatial resolution achieved.^58^

### Factors influencing tissue temperature and treatment success

Thermal ablation risks described in the previous paragraph can result from factors outside the ablative treatment, which strongly affect the growth of the thermal damage and thus the effectiveness/safety of the procedure. Such influencing factors are represented by the tissue properties and characteristics, such as electrical and thermal conductivity, dielectric permittivity, density, and heat capacity, as well as blood perfusion presence and amount.^44^ Their impact on the ablation outcomes is strongly related to the kind of source of ablation.

More specifically, in case of RFA, electrical conductivity represents the main parameter affecting the treatment success, since this technology of ablation relies on the current flow through tissue to cause adequate heating and cell death, which in turn is affected by tissue hydration status and its content of ions. Thus, tissues rich in water and ions such as liver would more effectively transmit current, whereas those with a lower content of water and ions such as lung, bone, and fat would have higher electrical impedance, making RFA less effective. Accordingly, the water content and permittivity of the tissues under consideration (which, for example, is approximately half for normal lung and bone tissue what it is for kidney) may necessitate equipment optimization.^16^ Additionally, as the ablation progresses, the tissue can become susceptible to dehydration and charring, phenomena which can increase tissue impedance to electric current flow. Similarly, LA is limited by tissue desiccation, charring, and carbonization, and it is heavily dependent on the optical properties of the target tissue, which vary with wavelength.^31^ On the contrary, MWA is not limited by tissue conductance since the propagation of MW energy is not dependent on the electric current, as previously detailed. Rather, MW propagation is described by the complex permittivity of the tissue and thus on the water content of the tissue, due to the MWA working principle.

The distribution of temperature inside the target lesion is also affected by the adjacent vasculature, which dissipates thermal energy, especially large blood vessels. Indeed, tissue blood flow represents the foremost factor limiting thermal ablation of tumors, since it acts as a heat sink either through large blood vessels or capillary-mediated perfusion, reducing the volume of tissue heated to target temperature. Thus, thermal ablation size and cytotoxic effectiveness decreases with proximity and size of adjacent vessels. Increased local recurrence rates of tumors adjacent to large vessels (especially those greater than 3 mm in diameter) demonstrate the significant effect of thermal energy sinks.^16^^,^^44^ Furthermore, in case of lung ablations, treatment outcomes are heavily affected by heat sinks from not only the surrounding pulmonary vasculature, but also the airflow due to respiration. Indeed, aerated lung tissue can act as an insulator, limiting the conductance of thermal and electric energy and causing an incomplete treatment of the tumor if not compensated by procedural techniques such as increased ablation time/power, or use of multiple applicators. On this regard, MWA, which does not rely on electric current conductance through tissue, has produced lung ablations 25% larger in the mean diameter compared with RFA. Therefore, developing methods to either decrease blood flow or increase heating efficacy may be vital to optimize the perivascular ablation lethality. In multiple studies, for example, the zone of coagulation is increased when hepatic blood flow is reduced by using arterial embolization techniques (e.g., balloons, coils, particles, or lipiodol agents).^16^

As a result, besides directing the choice of the most appropriate thermal ablation technique, the impact of the discussed influencing factors on the thermal dose delivered to the patient and thus on the outcomes of thermal ablation cannot be neglected. Against this background, temperature monitoring becomes imperative to control the ablation process also in terms of such external influencing factors, to avoid them from treatment impairment. In this scenario, temperature monitoring stands for a key enabler to investigate thermal ablation effects from different perspectives, i.e., not only to assess the influence of the treatment parameters on the treatment outcomes, such as thermal damage extension and temperature gradient, but also to deeply analyze and quantify phenomena such as blood perfusion, the impact of blood vessel proximity to the thermal source and type of treated tissue on the ablative treatment. On this matter, the following sections focus on FBGs as promising technique to control thermal ablation and on their leveraging during RFA, LA, and MWA experiments.

## Fiber optic sensors in thermal ablation

Fiber optic sensors (FOS) provide emerging techniques for temperature monitoring during thermal ablation treatments which are gaining a growing interest in the research field. Comparing them with respect to non-invasive techniques, it is worth noting that, although the clear advantages related to the absence of contact, image-based thermometry can be considered not mature enough to be used as a clinical tool for the monitoring phase of thermal ablation procedures, since the high costs, need of special calibration procedures, measurement artifacts, and other several limitations.^58^ In fact.

• Magnetic resonance imaging (MRI)-based thermometry, which is considered the current clinical gold standard among non-invasive thermometry, needs ad hoc designed sequences, and its thermal sensitivity depends on the types of tissue unless a proton resonance frequency shift technique is used. Moreover, potential concerns are related to the motion artifacts introduced by this technique and the fact that MR scanner can only be operated in conjunction with MR-compatible devices.^59^
• Computed tomography (CT)-based thermometry uses ionizing radiation and often requires multiple or continuous scan, hence the concern related to the dose which reaches the patient. Moreover, its thermal sensitivity is tissue-dependent, and only preliminary studies exist regarding its *in vivo* feasibility assessment. Other concerns are due to the poor involved temporal resolution. Furthermore, the repeatability of quantitative CT numerical measurements has not been guaranteed, which has hampered the clinical use of this method.^60^
• Ultrasound-based thermometry seems promising since the quick and convenient real-time visualization capabilities, but only in a temperature range up to about 50°C. In addition, thermal sensitivity depends on the nature of the tissue and unexpected variations of the acoustic properties of tissue can cause measurement artifacts.^61^

Therefore, a real control of the ablation procedure is beyond the present capability of the imaging systems, and invasive thermometric techniques are required.

Although invasive methods require the sensor to be inserted into the target tissue, they are much more cost-effective than imaging systems and overcome some of the previously described drawbacks. Currently, the most frequently used sensors in the clinical practice are thermocouples and thermistors. Indeed, in some commercially available models, sensors are embedded within the energy-delivering probe (e.g., StarBurst XL RFA device,^62^ Neuwave Medical Certus 140 MWA probe,^63^ both embedding several thermocouples within the thermal probe), thus minimizing the invasiveness of the procedure.

FOS are included in invasive thermometric techniques. However, with respect to conventional approaches, being smaller than their electrical counterparts, FOS do not alter the ablation pattern or exhibit hysteresis. Moreover, the possibility of using distributed or quasi-distributed sensing techniques allows the unmatched density of sensing points per units of length, providing sensing below 0.1 mm spatial resolution.^64^ Furthermore, FOS exhibit low-costs, immunity to electromagnetic interference, lightweight, flexibility, chemical and electrical inertness, remote operation, non-toxicity, and biocompatibility, all features which guarantee no immunity response from human defense system and a type of sensor intrinsically safe for the patient inertness.^65^ For these reasons, FOS meet the typical needs of biomedical applications such as thermal ablation and can substantially advance such tumor treatments providing real-time and spatially distributed biophysical sensing inside the target region.

### Toward multi-point temperature measurements: focus on fiber Bragg gratings

Among FOS, many authors report the use of fluorescence sensors, distributed sensors, and FBGs in both *ex vivo* and *in vivo* trials for temperature monitoring.^66^ However, FBGs stand out more than the other FOS in the pursuit of controlled thermal ablation, since they overcome the issues introduced by fluorescence and distributed sensors and combine their advantageous features, as highlighted in the following.

Fluorescence sensor technology is based on the relationship between fluorescence decay time of a rare-earth phosphor, which is located at the end of a fiber optic cable, and its temperature.^67^ The source is an UV lamp, which light pulses on the fiber tip excite the phosphor producing a fluorescence response resulting in visible light radiating back into the optical fiber.^68^ Since incoming and outgoing light beams are at different wavelength, no interference takes place. After the excitation, the fluorescent signal decays in a time function of the temperature of the phosphor itself with an exponential law. Therefore, the correlation between the decay time and the temperature of the fluorophore allows an estimate of the temperature at the fiber end. Furthermore, as the exponential decay is limited to few μs, fluorescence sensors typically have fast responses.

Several reports on the use of fluorescent sensors for temperature monitoring during thermal ablation treatments are in literature.^69^^,^^70^ Detection speed, accuracy, and the possibility of using a fiber-based probe as a disposable unit are attractive features for fluorescence systems.^58^ Other valuable features of this kind of sensors, beyond the typical benefits of FOS, are: i) a wide range of measurement, typically from −25°C to 300°C; ii) accuracy of 0.2°C; iii) independence on the intensity of excitation, iv) the chance to realize a multi-sensor system by sharing the pulsed excitation source among several channels.^71^ Moreover, since excitation light signal and fluorescent decay signal travel along the same optical path, the size of the fiber optic-based probe and sensing tip can be noticeably reduced. However, since fluorescence sensors allow performing single-point measurements, the need of multi-point or distributed measurements during thermal ablation treatments drove into the use of distributed and FBG-based sensors in several configurations.

Distributed sensing, often based on Rayleigh backscattering measurement, turns an entire fiber cable into a sensor, without discrimination between active and passive regions. Such systems achieve a minimum resolution of 20 μm with a maximum fiber length of 20 m and up to 1 Hz sampling rate. However, distributed optical sensors suffer from the back-reflection occurring at the fiber end which substantially overlaps the trace at the fiber tip.^64^ Such trace, indeed, is the most significant sensing information as this part of sensor is usually located in correspondence to the ablation tip. A possible solution is the introduction of the fiber into a microcatheter, which sustains the penetration while preserving the cleaving quality. Furthermore, an additional inconvenient showed by distributed sensors, i.e., a complex interrogation procedure, steers the choice of the suitable thermometry technique toward FBGs.^72^

FBGs encompass the simple spectrometer setup needs with multi-point measurement capabilities. Multiple sensors, indeed, can be placed within one single optical fiber obtaining an array of FBGs and permitting to monitor temperature in different points simultaneously without inserting more devices within the organ, which may increase the risk of bleeding. This quasi-distributed sensing approach is achieved by writing a number of sensors on the same optical fiber with a predetermined spacing. The capability for such multiplexing property has been exploited for the spatial mapping of the temperature distribution by using wavelength or time division multiplexing techniques to discriminate the temperature readings of each individual grating along the same fiber.^73^ Therefore, FBG arrays represent the most straightforward technique for inline temperature measurement with fiber optic technology.^64^ Their multiplexing property, together with the flexibility and small size typical of the optical fibers, makes FBGs – once placed in tiny needles – percutaneously insertable and usable also under endoscopic US guidance into the organ through natural orifices, minimizing their invasiveness. In addition, they are CT and MR compatible, and do not cause any measurement artifact due to the direct absorption of source energy.^66^ Furthermore, their good static and dynamic metrological accuracy and their short response time allow fast and accurate measurements.

By implementing particular fabrication strategies some properties of FBGs can be enhanced. The choice of the material to coat the gratings, for example, leads to more resistant sensors. This is the case of polyimide coating, which exhibit improved thermal properties over the standard acrylate coating, such as high-temperature resistance (i.e., up to 400°C).^74^ However, polyimide coating is hygroscopic and show swelling when the water molecules diffuse.^75^ This phenomenon generates a strain effect on the fiber and makes the polyimide-coated FBG sensitive to relative humidity, which variations are common in biomedical applications, potentially causing measurement artifacts if humidity does not represent the parameter of interest. Besides coating design, also FBG fabrication technique can affect the sensing features. Indeed, by using an array of FBGs fabricated on a drawing tower, it is possible to achieve a reduced spacing between each active region.^64^ Furthermore, during last years also chirped fiber Bragg gratings (CFBGs) have been investigated as sensors for monitoring the effects of thermal treatments.^76^^,^^77^ CFBGs allow achieving distributed sensing in a short active length with hundreds of sensing points. They make use of the same optical hardware as FBG sensors, employing the same interrogators, but achieving a theoretical resolution below 0.1 mm.

The risk involved by the use of FBGs and CFBGs during thermal ablation are mainly two:^78^ i) rupture of the sensor within the organ due to their fragility; ii) measurement error caused by respiratory movements (for *in vivo* applications), since the movements of the organ can strain the sensing elements causing periodic fluctuations of their output. In this regard, it is worth noting that artifacts due to the respiratory movements are related not only to applications in lung cancer treatments, but also to organs such as liver and pancreas, which also experience significant movements caused by patient respiration. The solution proposed in several works^65^^,^^79^^,^^80^ to overcome both these concerns is to embed the fiber optic within a needle, in such a way that both safety (i.e., the absence of rupture of the probe) and no measurement artifacts are guaranteed. Since the FBGs embedded in metal needles can suffer from self-heating once exposed to the thermal ablation source due to the thermal expansion contribution and to the light absorption, in case of LA, of metallic components, several investigators have exploited other materials, such as carbon fiber tubes employed such as protective needles.^81^^,^^82^ It is worth to note that, given the periodical nature of the respiratory artifact, the latter can be addressed also by implementing a filtering stage on the output signal with adequate bandwidth. In a clinical scenario, indeed, the mentioned bandwidth can be easily set since the respiratory frequency is known and typically controlled by the mechanical ventilator.^83^

However, compared to FBGs, CFBGs introduce an additional and significant drawback, which lies in the lack of a reliable technique for their detection in the spectrum domain in real-time operation. Simplified approaches substantially turn the CFBG into a few-point sensor, which does not offer relevant advantages over FBGs.^64^ Moreover, high-density distributed sensing based on optical frequency-domain reflectometry allows achieving resolution of 20÷200 μm with a tight performance trade-off (accuracy, resolution, and speed), but its *ex vivo* performances are hardly replicable for *in vivo* applications, unless special catheterizations are applied.

In conclusion, although CFBG sensors achieve a very high resolution, they are strongly dependent on the interpretation of the optical spectrum, which is suboptimal with the algorithms presented to date. Therefore, FBG arrays allow the most straightforward measurement and interpretation of data.

### Sensing mechanism of fiber Bragg gratings

**Figure 7:**
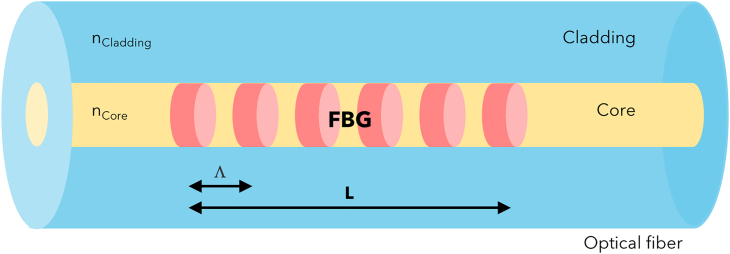
FBG structure Schematic of an FBG sensor inside the core of an optical fiber.

By definition, an FBG is the periodic perturbation of the refractive index (RI) realized in the optical fiber core along the propagation direction. Figure 7 reports the schematic of an FBG; usually the length L of the grating ranges from 1 mm to 1 cm, whereas the spatial period Λ is around 1 μm.

The FBG effect upon a light wave incident on the grating at an angle $\theta_{1}$ can be described by the grating equation:^84^

(Equation 1) $$n\;\sin\;\theta_{2}=n\;\sin\;\theta_{1}+m\frac{\lambda }{\Lambda }$$

being n the RI of the fiber core, λ the resonant mode wavelength, $\theta_{2}$ the angle of the diffracted wave, and m the diffraction order. Equation 1 is not capable of determining the wavelength at which the grating most efficiently couples light between two modes. Since $n\;\sin\;\theta =n_{eff}$ and the mode propagation constant is $\beta =\frac{2\pi }{\lambda }n_{eff}$, (1) can be rewritten as:

(Equation 2) $$\beta_{2}=\beta_{1}+m\frac{2\pi }{\Lambda }$$

For first-order diffraction, which usually dominates in a fiber grating, m=−1, where the minus sign describes modes that propagate in the −z direction, being z the propagation direction of the light along the optical fiber core. Therefore, $\beta_{2}=-\beta_{1}$ and, from Equations 1 and 2, λ can be obtained as follows:

(Equation 3) $$\lambda =\left(n_{eff,2}+n_{eff,1}\right)\Lambda$$

If the two modes of indexes $n_{eff,1}$ and $n_{eff,2}$ are identical, (3) becomes^84^^,^^85^:

(Equation 4) $$\lambda_{B}=2n_{eff}\Lambda$$

where $n_{eff}$ is the effective RI of the fiber core, and $\lambda_{B}$ represents the Bragg wavelength.

**Figure 8:**
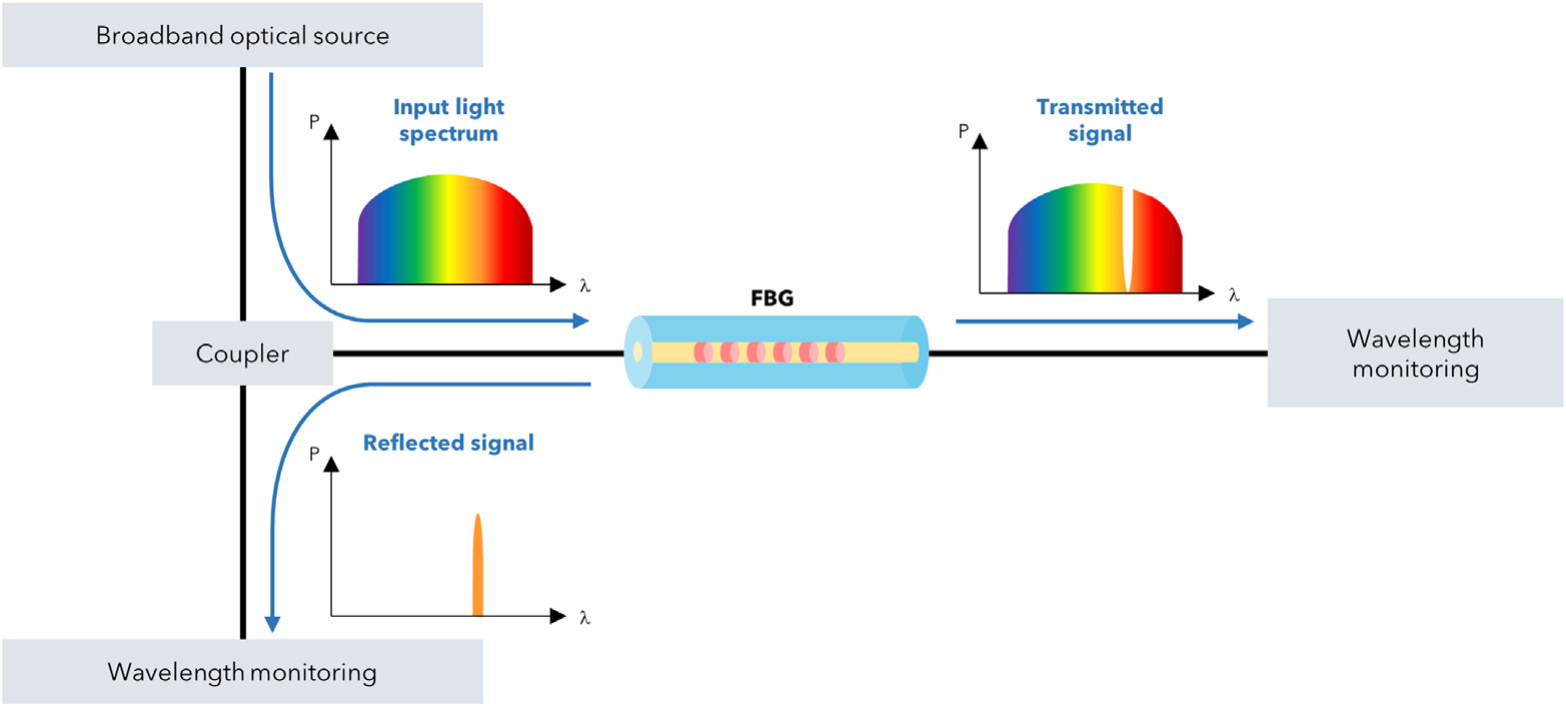
FBG working principle as band-stop filter Basic system of operation of an FBG sensor.

Therefore, by considering (4), the working principle of an FBG is as follows: in the presence of an incoming broadband light into the core, the Bragg grating reflects a narrow band of the input light, centered around the Bragg wavelength, while the other wavelengths go through the grating without any perturbations. Figure 8 reports the described principle of operation.

Equation 4 reveals that the main elements that influence and control the FBG properties are Λ and $n_{eff}$, which in turn are affected by two basic parameters, i.e., temperature and strain. Therefore, the FBG mechanism, in the elementary form, is based on observing the shift in $\lambda_{B}$ with changing in any of these parameters. However, FBGs can be utilized for various other sensing applications such as monitoring pressure, force, acceleration, viscosity, dynamic magnetic field, humidity, vibration acoustics, flow, and so forth by employing suitable transduction techniques.^68^

The shift in $\lambda_{B}$ due to strain and temperature changes is given by:^86^

(Equation 5) $$\Delta \lambda_{B}=2\left[\Lambda \frac{\partial n_{eff}}{\partial L}+n_{eff}\frac{\partial \Lambda }{\partial L}\right]\Delta L+2\left[\Lambda \frac{\partial n_{eff}}{\partial T}+n_{eff}\frac{\partial \Lambda }{\partial T}\right]\Delta T\text{,}$$

The first term in Equation 5 represents the strain effect on an optical fiber (i.e., $\Delta \lambda_{B,s}$), which corresponds to a change in the grating spacing and the strain-optic induced change in the RI, and can be expressed as:

(Equation 6) $$\Delta \lambda_{B,s}=\lambda_{B}\left\{1-\frac{n^{2}}{2}\left[p_{12}-\nu \left(p_{11}+p_{12}\right)\right]\right\}\varepsilon_{z}\text{,}$$

where $p_{11}$ and $p_{12}$ are the components of the strain-optic tensor, n is the RI of the core, ν is the Poisson’s ratio, and the strain is $\varepsilon_{z}=\delta L/L$. For a typical optical fiber, $p_{11}=0.113$, $p_{12}=0.252$, ν=0.16, and n=1.482, so that the expected sensitivity at approximately 1550 nm is a change of 1.2 p.m. as a result of applying 1 με to the grating.

The second term in Equation 5 represents the temperature effect on an optical fiber (i.e., $\Delta \lambda_{B,s}$). This fractional wavelength shift for a temperature change ΔT can be written as:

(Equation 7) $$\Delta \lambda_{B,T}=\lambda_{B}\left(\xi +\alpha \right)\Delta T\text{,}$$

where the quantity $\xi =\left(1/n_{eff}\right)\left(dn_{eff}/dT\right)$ represents the thermo-optic coefficient (approximately equal to 8.6⋅10^−6^°C^−1^ for the germanium-doped silica core fiber), whereas α=(1/Λ)(dΛ/dT) is the thermal expansion coefficient for the fiber (approximately, 0.55⋅10^−6^°C^−1^ for silica).^87^ The term (ξ+α) can be identified with the thermal sensitivity $S_{T}$ of the FBG, which expected value at 1550 nm and in the temperature range from approximately 30°C–100°C has a typical value of 6.5⋅10^−6^°C^−1^.^86^

It is relevant to observe that, if both strain and temperature changes are involved, the measurement of the shift of $\lambda_{B}$ cannot be unequivocally correlated with either parameter. One approach to overcome this limitation is to determine temperature and strain from two independent measurements, each with different known temperature and strain coefficients, e.g., two FBGs with different fiber compositions or with very different periods.^88^ However, this approach suffers from the additional complexity due to the need to interpret two diverse transfer functions from each type of sensor.^89^ Alternatively, an FBG for which the effect of a parameter (strain or temperature) is nullified can be employed, thus enabling a pure measurement of the second parameter. In this case, if only one parameter needs to be measured, then such a single isolated grating may be sufficient. However, if both parameters are of interest, then a second sensor is required. While temperature isolated gratings are commercially available, the isolation of the FBG from strain in its surroundings can be obtained by adopting embedding methods, e.g., terminating the fiber at the end of the FBG and embedding the fiber end in a hollow capillary like the encapsulation within needle discussed in the previous paragraph. Due to its freedom inside the capillary, in this configuration no strain will act on the sensor. If the FBG is not located at the end of the fiber, several embedding solutions have been implemented to achieve axial strain isolation. One possible way is to place the fiber in a capillary in such a manner that the encapsulated part of the fiber, containing the FBG, is bent in an arc, while the fiber is fixed only in correspondence of the capillary ends.^88^ In this way, when the capillary is stretched, the fiber merely straightens with no significant axial strains involved. However, with this approach, a capillary with a large diameter is needed, since it must accommodate the curved fiber.

## Results of thermal ablation controlled by fiber Bragg gratings

In this Section, the literature studies on the use of FBG sensors in RFA, LA, and MWA procedures are reported. Each of the following subsections focuses on a specific thermal ablation treatment and highlights addressed issue, investigated tissue model, ablation probe and settings, and configuration of the FBGs for each reviewed study.

Since the first experiments proposed by Rao et al. in 1997 [91], the use of FBGs for monitoring thermal treatments has gained widespread interest [79]. The first *in vivo* trial was carried out in 2000 by Webb et al. on diseased livers and healthy kidneys of rabbits undergoing hyperthermia [92]. This work paved the way for the numerous studies focusing on the use of FOSs during *in vivo* trials. Applications of arrays of FBGs for performing temperature measurements with high resolution have been investigated in several *ex vivo* animal models (e.g., kidney [93], liver [24], pancreas [94], lung [95]) and *in vivo* ones (e.g., pig [67]).

Besides distinguishing among the specific techniques of thermal ablation which have been investigated in literature, the studies on FBGs for thermal ablation control can be classified also according to the specific aspects and issues addressed by different authors. Therefore, this Section aims to provide an overview of the state of the art of the temperature monitoring through FBGs during RFA, LA, and MWA focusing on the different facets of such kinds of treatments and on the various conclusions which can be inferred from the temperature measurements.

### Fiber Bragg gratings for radiofrequency ablation monitoring

Temperature distribution during RFA is the most widely studied due to the extensive use of the RFA technique for tumor treatment.^58^ Several investigators studied the design and prototyping of a temperature monitoring system during RFA. Table 2 collects the state-of-the-art regarding the use of FBG sensors during such technique of thermal ablation. The reported articles are grouped by the issues addressed by the authors, and, for each group of works investigating the same points, a chronological order has been followed. The key aspects for each listed work are highlighted, i.e., explored tissue model, type of RF applicator, treatment settings, and configuration of the involved FBGs. In the following, Table 2 is illustrated by deepening the reported works.

**Table 2:** Key details of the main FBG-based temperature monitoring systems for RFA proposed in literature, organized by the addressed issue

Addressed issue	Tissue model	RF probe and treatment settings	Experimental configuration of FBG sensors	Reference
Experimental evaluation of the temperature profiles as the function of time inside the target tissue	*Ex vivo* swine liver	Power = 20 W Exposure times = 165÷310 s RF generator frequency = 480 kHz	1 array of 5 FBGs, each one 5 mm long and 5 mm spaced, installed on the applicator	Tosi et al.^90^
	*Ex vivo* swine liver	Monopolar hooked RF needle, *MRI compatible StarBurst®XL Electrosurgical Device* Power = 250 W Exposure time = 380 s RF generator frequency = 460 kHz	Two needles embedding: - 1 array of 6 FBGs, each one 3 mm long and 2 mm spaced, in parallel with the applicator at 8.6 mm from it - 1 array of 3 FBGs, each one 1 mm long and 2 mm spaced, in parallel with the applicator at 6.4 mm from it	Saccomandi et al.^91^
	*In vivo* swine liver	Monopolar RF needle, *Star Electrode Fixed type, STARMed®* Power = 80 W Exposure time = 380 s RF generator frequency = 480 kHz	Needle-probe embedding 1 array of 3 FBGs, each one 1 mm long and 2 mm spaced, placed in parallel with the applicator at 11 mm from its tip	Saccomandi et al.^66^
	*Ex vivo* bovine liver	Monopolar RF needle, *Cool-tip™ RF Ablation System, Covidien* Power = 25÷100 W Exposure time = 720 s RF generator frequency = 480 kHz	Needle embedding 1 array of 4 FBGs which are 10 mm spaced, placed at 10 mm from the center of the applicator	Leng Lee et al.^92^
Experimental evaluation of the spatial maps of temperature measured inside the target tissue	*Ex vivo* bovine liver	Bipolar applicator, 4 RF electrodes, *Habib 4x Bipolar Resection Device, Angiodynamics* Power = 125 W RF generator frequency = 500 kHz	5 arrays, for a total of 27 FBGs, embedded inside carbon fiber needles and attached to the applicator, at distances from the center of the electrodes ranging from 0 to 10.5 mm	De Vita et al.^93^
Experimental analysis of blood perfusion contribution to the treatment outcomes	*Ex vivo* bovine liver and kidney			De Vita et al.^94^
Experimental analysis of the influence of the RF applicator depth inside the tissue on the treatment outcomes	*Ex vivo* swine liver			Palumbo et al.^82^
Experimental evaluation of the treatment enhancement induced by using NPs	*Ex vivo* swine liver	Monopolar RF needle Power = 50 W Exposure times = 119÷172 s RF generator frequency = 450 kHz	3 arrays of 5 FBGs, each one 5 mm long and 5 mm spaced, located at 0, 5 mm, and 10 mm from the applicator	Jelbuldina et al.^95^
	*Ex vivo* swine liver		1 array, 7.61 cm long, of 16 FBG, alongside the applicator	Ashikbayeva et al.^96^

**Figure 9:**
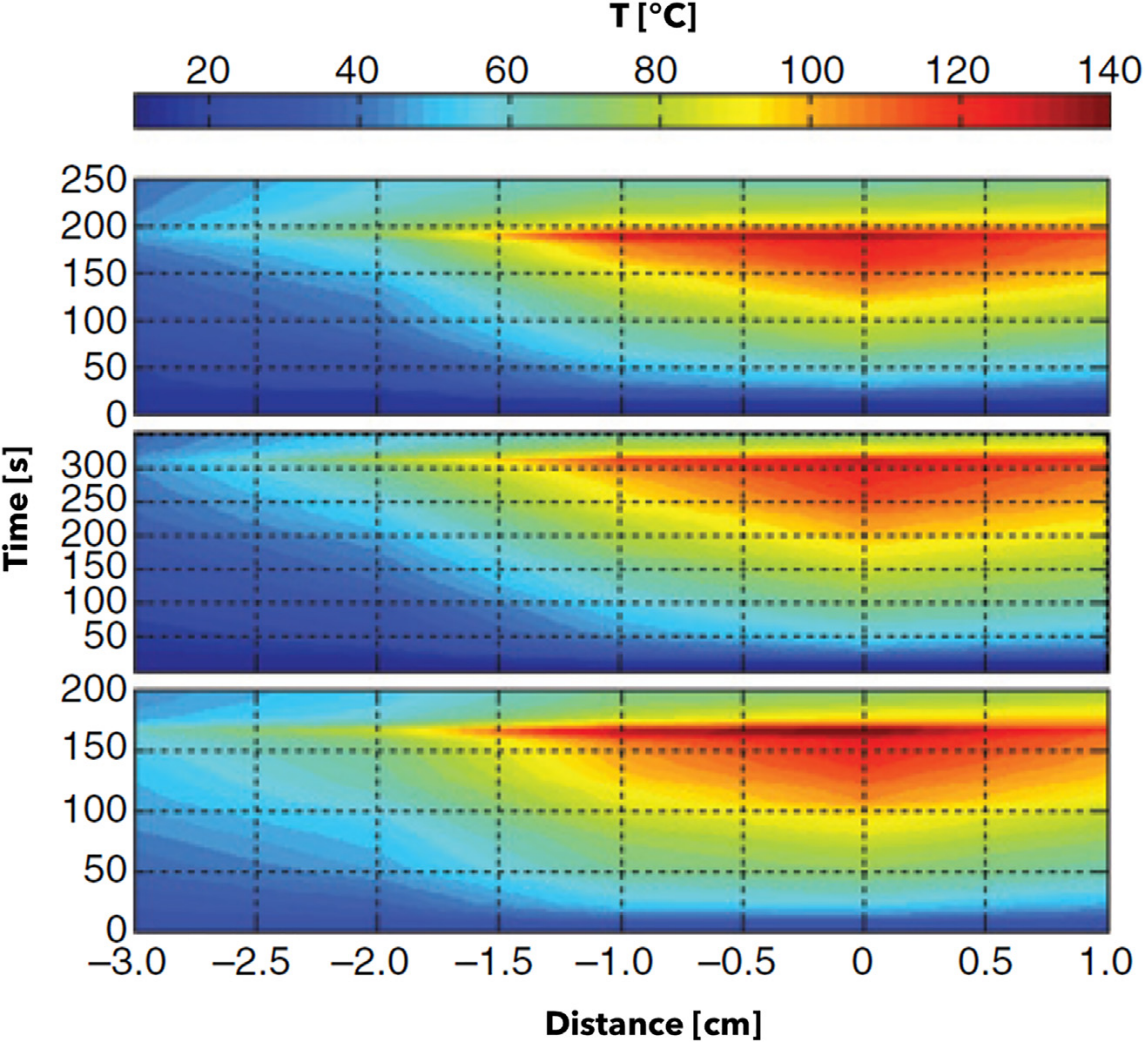
Examples of FBG-recorded thermal maps as the function of distance during RFA Temperature distribution along the array axis recorded during three different RFA discharges performed at 20 W of power. Reprinted with permission from.^90^ © Wiley.

Starting from the basic use of FBGs for RFA, i.e., with the aim of measuring the temperature profile as the function of time inside the target tissue, Tosi et al.^90^ installed a multi-point FBG-based probe on a device for RFA to provide quasi-distributed thermal pattern measurements and plot thermal maps as the function of time along the applicator axis during *ex vivo* experiments on swine liver (see Figure 9).

Still aiming to an experimental evaluation of the temporal trends of temperature, Saccomandi et al. performed: i) CT-guided RFA of *ex vivo* swine livers by using two custom-made thermal probes embedding a total of 9 FBGs (2 sensors/cm and 3 sensors/cm for the two FBG arrays, respectively), in addition to the 5 thermocouples embedded in the umbrella-shaped RF probe;^91^ ii) US-guided RFA of *in vivo* swine liver, drawing on a needle-like probe embedding an array of three FBGs.^66^ The measurement errors less than 3°C assessed in the latter work and induced by the breathing movements encourage the use of FBG-based probes in the clinical scenario. Similarly, Lee et al.^92^ reported on temperature measurements during CT-guided RFA, investigating the correlation between the shift of the CT number and the temperature variation inside the tissue of *ex vivo* bovine livers, finding a negative linear relationship. In addition to the measurements of the temperature trends inside the lesion during the time, more recently FBGs have been exploited to reconstruct the spatial maps of temperature within the tissue volume containing the applicator.

In^93^ a dense configuration of FBGs is proposed, in which the five arrays of sensors are embedded inside carbon fiber needles attached to a commercial RF applicator by means of an *ad hoc* realized support. Such FBG-based probe provided the 2D thermal map in the plane between the RF electrodes and containing the arrays of FBGs in real-time. The sensorized probe has been investigated also to deepen other aspects of RFA treatments. Indeed, the great potential of the FBGs in terms of spatial and temperature resolution, high enough to follow the temperature gradient inside the thermal lesion, allows to employ them also for examining the blood perfusion contribution to the RFA outcomes by emulating the blood flow in *ex vivo* models.^94^

**Figure 10:**
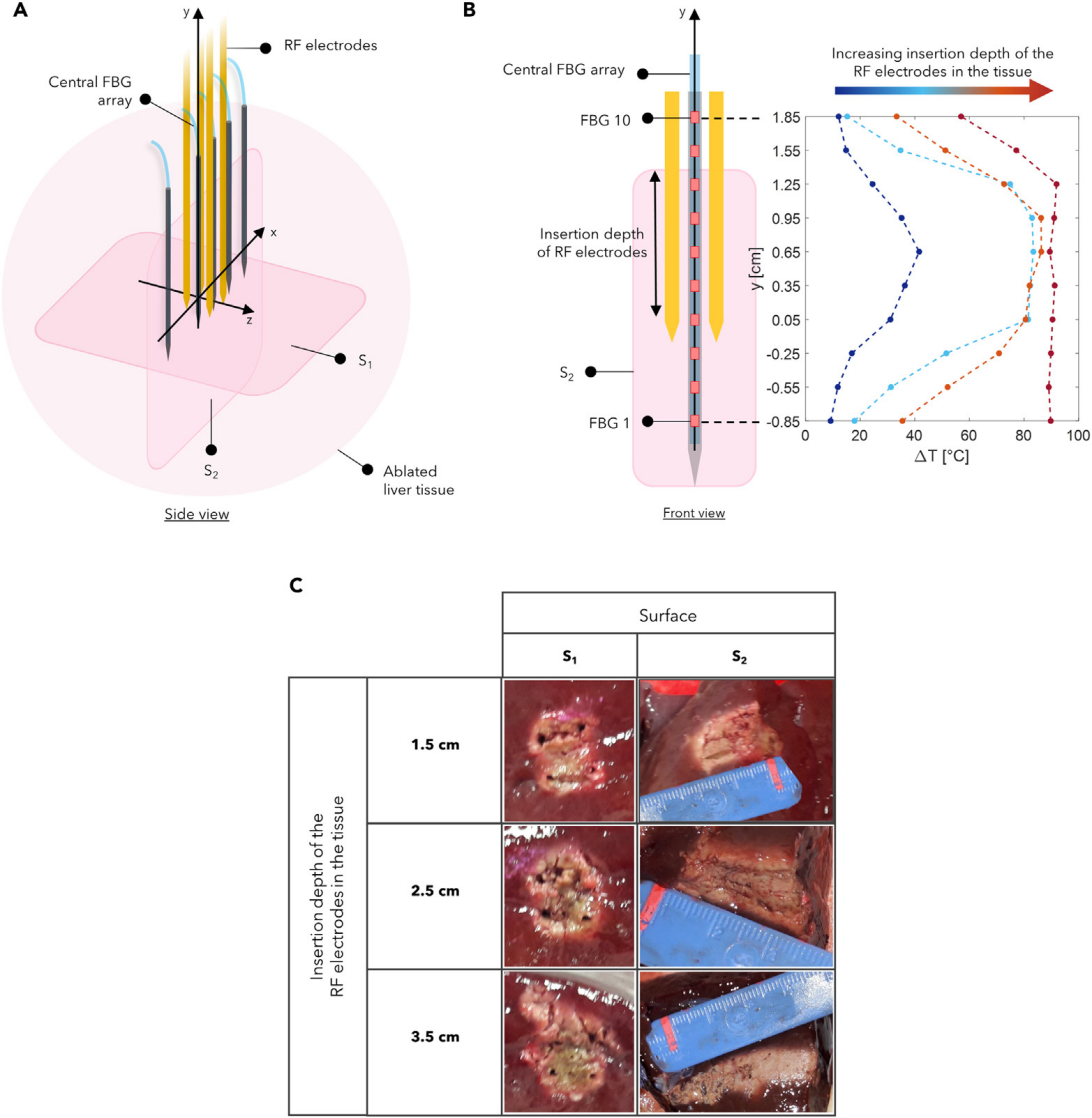
Effects of different insertion depths of the RF probe inside the tissue (A) Schematic of RF electrodes and in-needles FBGs inside the tissue undergoing RFA. (B) Focus on the FBG array in the middle of the RF electrodes and temperature profiles recorded by its ten FBGs as the function of the probe depth. (C) Photos of the ablated tissue in correspondence of the two for three probe depths inside the tissue. Adapted from.^82^ Under a Creative Commons license.

Further RFA *ex vivo* tests with such FBG-based probe but aiming to a different purpose are reported in,^82^ which assesses that the depth of the RF applicator affects the ablation results in terms of maximum recorded values of temperature, spatial distribution of temperature increase inside the tissue, and time duration of the RF discharge by the used generator, endowed with an impedance-based control of power delivery. The network of FBGs, indeed, recorded a significant increase of all the mentioned parameters by increasing the insertion depth of the applicator inside the tissue, as reported in Figure 10.

The schematic of RF probes and FBGs embedded in needles inside the tissue undergoing RFA is represented in Figure 10A, highlighting the sections S_1_ and S_2_, whereas results are reported in Figures 10B and 10C, where temperature profiles recorded by the central array as and photos of the ablated tissue as the function of the probe depth are shown, respectively.

Another RFA prospect which FBGs allow investigating is the evaluation of the enhancement of the treatment outcomes induced by involving NPs. In this context, FBGs become essential to analyze the spatial distribution of temperature, rather than simply observe the damaged tissue via imaging or using a colorimetric phantom, in order to evaluate the procedure trend from the heat propagation point of view.

In,^95^ indeed, the authors reported about a sensing system based on a network of 15 FBGs for the thermal profiling of RFA enhanced by ferromagnetic NPs, exploring three values of concentrations of NPs (c_NPs_). By reconstructing the thermal profiles and maps as the function of time inside the tissue, they discovered an improvement of the heat delivering to the tumor boundaries by using moderate c_NPs_ (i.e., 5 mg/mL) and, on the contrary, a limited heat spread by involving a large c_NPs_ (i.e., 10 mg/mL). Similarly, in^96^ the effect of iron oxide magnetic NPs on the temperature increment during RFA has been analyzed by involving an array of 16 FBGs and considering two different solvents and ten values of concentration, revealing even in this case the best outcomes when 5 mg/mL is employed.

Besides the thermal analysis by means of FBGs, fiber optic sensors have been used also for pressure monitoring during RFA. Tosi et al.,^97^ indeed, developed a fiber optic probe embedding, together with a proximal FBG, an extrinsic Fabry-Perot interferometry to provide dual pressure and temperature measurements during *in vitro* and *ex vivo* tests on swine liver undergoing RFA. Indeed, the vaporization of the liquids contained in the tissue, caused by the exceeding of 100°C, generates a pressure increase in the heating point which can induce the dislodgement and scattering of malignant cells around the ablated tumor. This is the reason why also pressure monitoring can prove useful. Moreover, FBGs have been used also as strain sensors in this application with a view to detect the position of the RF applicator within the target tissue, combining MRI with the FBG measurements.^98^

### Fiber Bragg gratings for laser ablation monitoring

One of the first applications of FBGs for thermometry in LA was presented by Ding et al. in 2010, who developed a distributed FBG sensor with a length of 10 mm, encapsulated within a glass capillary, for temperature monitoring in an *ex vivo* liver undergoing the treatment.^99^ Since then, several studies have been carried out aiming to investigate LA implications and outcomes from different viewpoints. Table 3 lists the main addressed issues about LA and the related works from the literature, following the same scheme implemented in Table 2.

**Table 3:** Key details of the main FBG-based temperature monitoring systems for LA proposed in literature, organized by the addressed issue

Addressed issue	Tissue model	Laser probe and treatment settings	Experimental configuration of FBG sensors	Reference
Experimental analysis of the relationship between CT numbers and temperature variation	*Ex vivo* swine pancreas	ND:YAG laser (D = 300 μm, λ = 1064 nm), *Laser DEKA Medical Electronics®* Power = 3 W Exposure time = 120 s	4 arrays of 3 FBGs, each one 1 mm long and 2 mm spaced, perpendicular to the applicator and at distances from its tip ranging from 4 mm to 30 mm	Schena et al.^100^
Numerical and/or the experimental assessment of FBG-based sensing systems with different characteristics	Agar phantom	- Non-medical laser system for measurements by means of thermographic camera: Power = 3 W Exposure time = 180 s - Resistor mimicking the laser applicator for measurements by means of thermocouples and FBGs: Power = 7.5 W	- 1 FBG 15 mm long, perpendicular to the resistor axis, at 8 mm from it - 1 array of 3 FBGs, each one 1 mm long and 4 mm spaced, perpendicular to the resistor axis at 4 mm, 8 mm, and 12 mm from it	Gassino et al.^101^
	Agar phantom and *ex vivo* swine pancreas	Laser diode system (D = 300 μm, λ = 1064 nm), *LuOcean Mini 4, Lumics* - Settings for tests on phantom: Temperature threshold = 12°C Exposure time = 60 s - Settings for tests on pancreas: Temperature threshold = 40°C Exposure time = 120 s	- 4 commercial arrays of 10 FBGs, each one 1 mm long and a 1 mm spaced, in parallel to the applicator, at 2 mm from it - 4 custom-made arrays of 25 FBGs, each one 0.9 mm long and a 0.1 mm spaced, in parallel to the applicator, at 2 mm from it - 8 custom-made arrays of 40 FBGs, each one 0.19 mm long and a 0.01 mm spaced, in parallel to the applicator, at 4 mm from it	Morra et al.^102^
Numerical and experimental evaluation of thermal damage and profiles and maps of temperature as the function of time and space	*Ex vivo* swine liver and urethra	- Tests on liver: Laser diode (λ = 980 nm), *Apollo Instrument* Power = 3÷5 W Exposure time = 10÷120 s - Tests on urethra: Customized diffusing LITT applicator (D_core_ = 600 μm, λ = 980 nm) equipped with a glass tip cap (D_out_ = 1.4 mm) Power = 4 W Exposure time = 72 s	1 FBG 5 mm long, in parallel to the applicator in correspondence of the middle of the diffuser, at various distances from it, ranging from 0 to 3.5 mm	Pham et al.^103^
Development of a laser probe for customized irradiation and integrating FBG sensors for temperature measurements	Agar phantom and *ex vivo* animal liver	Laser diode system (λ = 915 nm). Dual cladding fiber, protected in a quartz capillary, as probe integrating a customized laser irradiation pattern and 2 FBGs (D_core_ = 20 μm, D_cladding,int_ = 400 μm). Probe surface is modified by means of a CO_2_ laser to produce a uniform radiation pattern. - Settings for tests on phantom: Power = 2 W Temperature threshold = 50°C - Settings for tests on liver: Power = 2÷4 W Energy = 300 J	- 2 FBGs, 5 mm long and separated by 3 mm, integrated inside the laser probe - 3 external FBGs in parallel to the probe, one immediately close to it, one at 0.5 cm and one at 1 cm from it, the latter used only during the tests on phantom	Gassino et al.^34^
Closed-loop temperature control basing on FBG measurements	*Ex vivo* bovine liver	Laser diode system (D = 800 μm, λ = 450 nm). Probe made of laser fiber and FBG sensor inside a sealed-end stainless-steel tube - Settings for probe characterization: Power = 3÷5 W Exposure time = 24 min Temperature thresholds = 0÷120°C - Settings for tract ablation: Power = 3÷5 W Exposure time = 90 s Temperature threshold = 150°C Speed of probe displacement = 1 mm/s	1 FBG 2 mm long, integrated inside the laser probe	Abd Raziff et al.^104^
	*Ex vivo* swine liver	Laser diode system (D = 440 μm, λ = 808 nm) and collimator for contactless superficial ablation at 7 cm from tissue. Power = 5 W Exposure time = 90 s	3 arrays of 40 FBGs, each one 1.19 mm and 0.01 mm spaced, perpendicular and centered with respect to the applicator and at 2 mm from each other	Korganbayev et al.^105^
Experimental evaluation of the treatment enhancement induced by using NPs	*In vitro*	Laser diode system (D = 300 μm, λ_1_ = 808 nm, λ_2_ = 940 nm, λ_3_ = 1064 nm) Power = 2.5 W Exposure time = 120 s	2 arrays of 25 FBGs, each one 0.9 mm long and 0.1 mm spaced, in parallel to the applicator, at 2 mm from it	Maor et al.^106^
Experimental analysis of blood perfusion contribution to the treatment outcomes	*Ex vivo* swine lungs	ND:YAG laser (D = 300 μm, λ = 1064 nm), *Laser DEKA Medical Electronics®* Power = 2 W Exposure time = 5 min	7 arrays of 5 FBGs, each one 1 mm long and 1 mm spaced, in parallel to the applicator and spatially arranged in a network around it, at distances ranging from 2 mm to about 4.5 mm from applicator tip	De Vita et al.^107^
Experimental assessment of measurement artifacts of FBGs due to strain variations	*Ex vivo* swine liver	ND:YAG laser (λ = 1064 nm), *Laser DEKA Medical Electronics®* 10 cycles-test Power = 2 W and 4 W Exposure time = 5 s per cycle Cooling time = 30 s per cycle Motorized ventilation not during LA = 10 breaths/min	- 1 bare FBG, 1 cm long, and 1 FBG fixed within a needle by means of epoxy adhesive, 1 cm long, each one perpendicular to the applicator, on either side, at distances ranging from 3 mm to about 8 mm from applicator tip - 1 bare FBG, 1 cm long, and 1 FBG encapsulated inside a needle by means of a thermal paste, 1 cm long, each one perpendicular to the applicator, on either side, at distances ranging from 3 mm to about 8 mm from applicator tip	Polito et al.^79^
	*Ex vivo* swine lungs	ND:YAG laser (D = 300 μm, λ = 1064 nm), *Laser DEKA Medical Electronics®* Power = 3 W Exposure time = 120 s Manual ventilation during LA = 12 breaths/min in the first 100 s	3 arrays, each one of the 7 FBGs 1 mm long and 2 mm spaced, at a distance from the applicator of 8 mm, 12 mm, and 30 mm, respectively	De Tommasi et al.^83^

In the first work reported in Table 3, authors employ several arrays of FBG sensors to experimentally validate CT-based thermometry, demonstrating the dependence of CT numbers on temperature variations occurring during LA on *ex vivo* animal pancreas.^100^

Other works investigate the dependence of FBG-based measurements on the temperatures at stake and on the morphological characteristics of the employed sensors. On this matter, Gassino et al.^101^ reported on the use of FBGs having different lengths in non-uniform temperature conditions, reproducing the LA treatment in a phantom which mimics the liver behavior from the optical and thermal points of view. The recorded temperature measurements are affected by an error strongly dependent on length of the FBG, proximity to the highest thermal gradient inside the phantom, and temperature value. Results highlight the need of FBGs shorter than 3 mm for more accurate measurements of the maximum temperatures inside the target volume and of a tradeoff between measurement error due to the grating length and error due to the grating spectral response, which in case of short FBGs presents large peaks and smaller reflectivity, thus involving worse temperature measurement capabilities.

Morra et al.,^102^ instead, compared FBG performances with Rayleigh scattering-based distributed sensing during contactless LA on agar gel phantom, assessing that, despite the spatial resolution improvements introduced by distributed sensors, signal-to-noise ratio over time and temperature standard deviation over temperature show better performances of FBGs. Moreover, the authors reported about experiments of temperature monitoring and mapping during contact LA on *ex vivo* swine pancreas by means of several arrays of FBGs having different characteristics (i.e., number and length of the sensors and of spacing among the sensors inside the same array, as detailed in Table 3), comparing the impact of their spatial resolution on the measurement capabilities of the temperature distribution.

**Figure 11:**
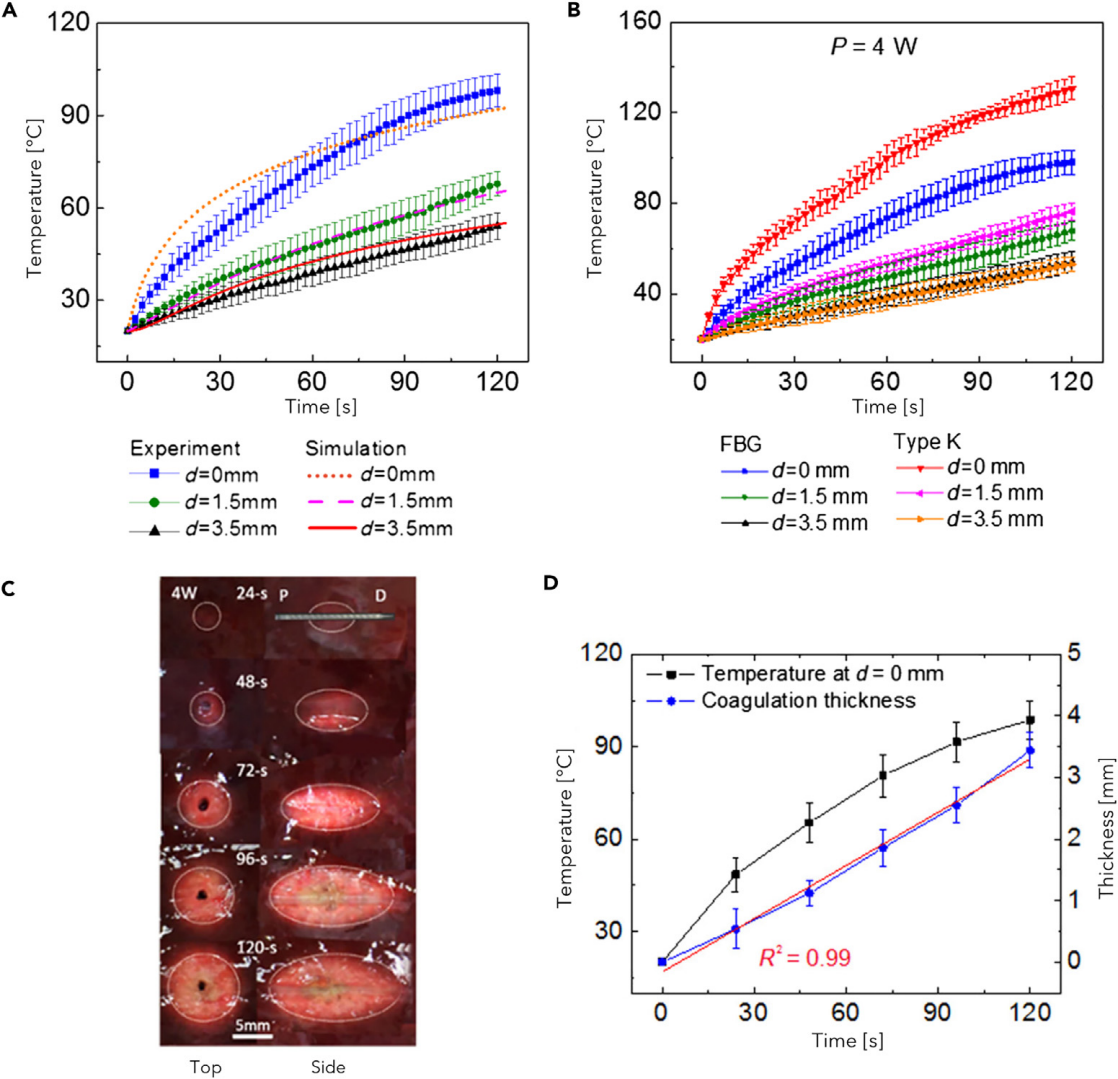
Results of LA performed at 4 W on *ex vivo* swine liver (A) Simulated and FBG-measured temperatures at 0, 1.5, and 3.5 mm from the diffuser surface. (B) Temperatures measured by FBG and type K thermocouple at 0, 1.5, and 3.5 mm from the diffuser surface. (C) Photos of the damaged tissue in correspondence of the transversal and side sections (top and side views, respectively) of the applicator axis at various irradiation times. (D) FBG-measured temperatures and corresponding transversal coagulation thickness at 5 mm from the diffuser end. Reprinted with permission from.^103^ © SPIE.

Together with temperature monitoring, thermal damage assessment assumes a relevant role to verify the degree of tissue coagulation caused by LA. In this regard, aiming at monitoring both temperature distribution and thermal damage, Pham and colleagues performed numerical and experimental analysis of temperature profiles during diffuser-assisted LA on *ex vivo* swine liver and urethral tissue by employing an FBG and a thermocouple besides numerical computations.^103^ They found a good agreement between simulated and measured temperatures at various distances from the applicator and setting parameters (see Figure 11A) and demonstrated a temperature overestimation of about 50% by the thermocouple when placed at 0 mm from the applicator (see Figure 11B), due to direct light absorption by the metal tip of the thermocouple and the consequent self-heating.

Moreover, simulations of temperature distributions and thermal damage at the end of the treatment and cross-sectional histology images at various positions along the applicator axis allowed to relate temperature measurements, light intensity, and coagulation shape and thickness, and to compare these parameters for the two kinds of tissue under test. In this regard, Figure 11C reports the photos of the thermal damage during hepatic LA at 4 W at various irradiation times, whereas Figure 11D shows the comparison between the temperatures recorded by the FBG and the corresponding transversal coagulation thickness at 5 mm from diffuser end, highlighting that both increase with irradiation time, but the extent of the tissue coagulation follows a linear profile.

Another addressed issue concerns the integration of the laser applicator with FBG sensors to obtain an all-optical probe able to deliver the laser power for the treatment and, at the same time, monitor it in terms of temperature. In^34^ the development, characterization, and validation of a sensorized laser applicator embedding two FBGs are presented. The proposed probe lies in a double cladding fiber to guide the FBG signal through the core, for temperature measurements, and the high-power beam used for ablation through the inner cladding. Furthermore, a laser assisted micro-patterning of the fiber tip stripped of the outer cladding was employed to deliver a customized irradiation pattern, enabling the matching of different tumor sizes and shapes.

In,^104^ besides the proposal of the laser applicator integration with an FBG sensor, in this case simply obtained by inserting both fibers for laser and sensor inside a sealed-end stainless-steel tube also working as a shield for the FBG from mechanical strain and risk of breakage, a closed-loop control based on proportional-integral-derivative (PID) system is implemented to adjust the laser power according to the temperature measurements. The system was tested on *ex vivo* bovine liver tissue during the withdrawal of the probe to investigate the impact of tract LA procedure to tissue coagulation, accompanying the temperature measurements of the internal FBG with an external chirped grating during the probe characterization and with histological analysis of the ablated tissue at the end of the treatment. A different kind of closed-loop control of LA is investigated in,^105^ where the authors proposed an ON–OFF logic to set the operating mode of the laser source according to the temperature measured by means of highly resolved FBG arrays fabricated with the femtosecond point-by-point writing technology. Although PID control results more effective to reach an adaptive control system, avoiding the oscillations of the temperature profile inside the lesion around the threshold value involved by an ON-OFF strategy, this closed-loop algorithm relies on the temperature map reconstructed in real-time by means of dense FBG arrays.

Indeed, the experimental FBG data and the interpolation along and between their axes enabled the software to identify the maximum temperature position and, from there, to select the radius of the region to be automatically controlled. Highly dense FBG arrays having characteristics analogous to those employed in^102^^,^^105^ have been also used to investigate the heating efficiency of combining thermal response induced by LA with theranostic properties of inorganic NPs,^106^ designing a thermal therapy approach capable of involving lower laser power, shorter exposure time and thus less collateral damage to surrounding healthy tissues with respect to those required in conventional therapy.

**Figure 12:**
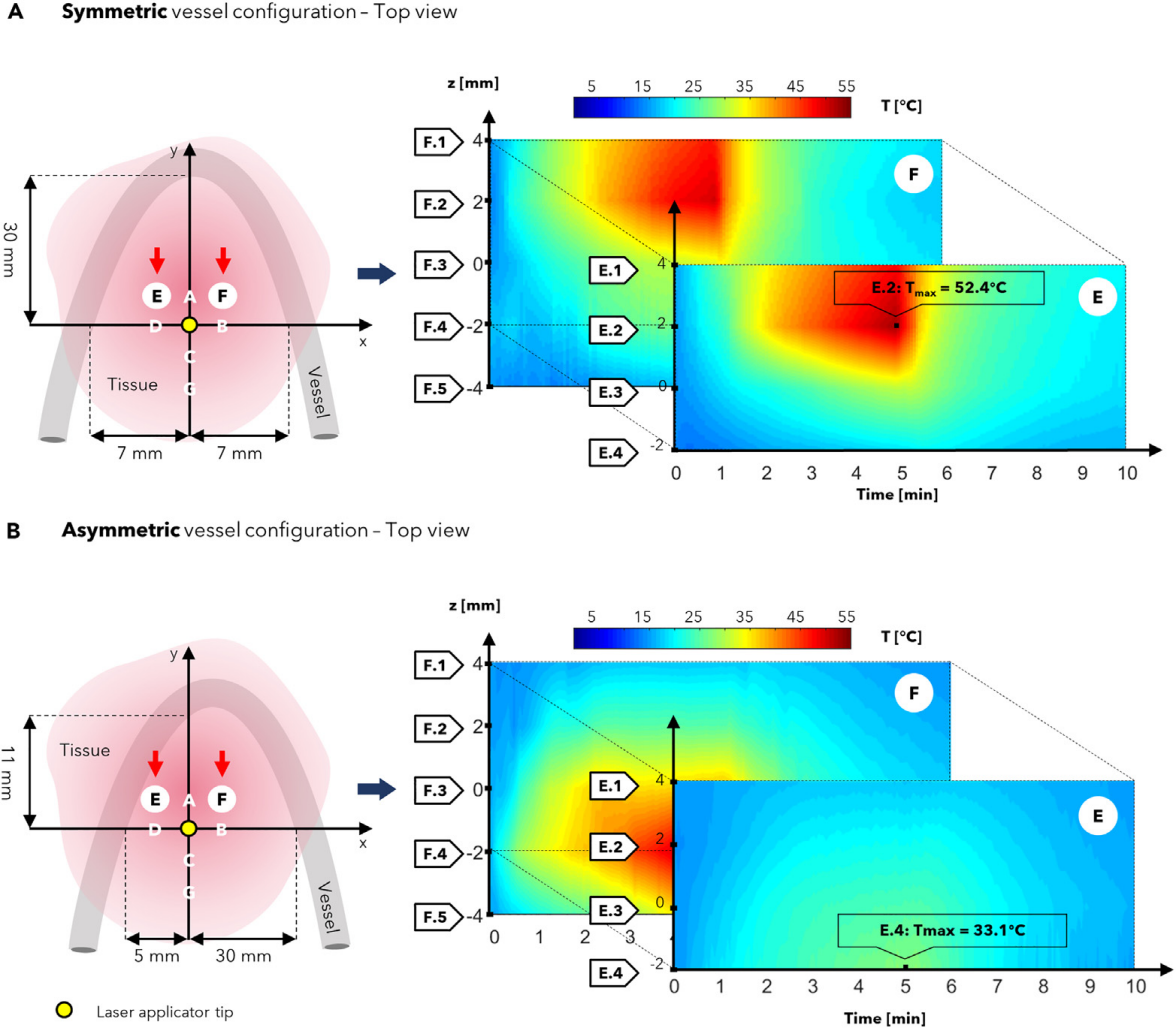
FBG-recorded thermal maps during LA on *ex vivo* swine liver demonstrating the cooling effect caused by the blood vessel (A) Symmetric vessel: top view of the configuration of sensors, laser tip, and vessel (on the left), and temperature maps recorded by arrays E and F as the function of time. (B) Asymmetric vessel: top view of the configuration of sensors, laser tip, and vessel (on the left), and temperature maps recorded by arrays E and F as the function of time. Adapted from.^107^ Under a Creative Commons license.

One of the most impactful factors affecting thermal ablation, as previously mentioned in Paragraph 3.2, is represented by blood perfusion, still under study since the difficulties in experimentally emulating real perfused blood vessels in the *ex vivo* scenario. In case of LA of liver, the consideration of perfusion presence and related heat sink effect becomes crucial due to the typically small size of an LA-ablated lesion and the high hepatic vascularization rate. FBGs play a major role also in this scenario, allowing to observe and quantify the influence of blood perfusion on the treatment outcomes, as demonstrated in.^107^ In this work, indeed, several arrays of FBGs have been distributed inside the target portion of liver undergoing LA to map the temperature variation around the applicator and a blood vessel surrogate. By the analysis of the recorded temperature profiles and maps for different spatial configurations of applicator, sensors, and vessel, a decrease in the maximum temperature variation up to 65% has been measured within 2 mm from the laser tip, as recorded by the arrays E and F which maps are reported in Figure 12, assessing the cooling effect caused by perfusion, the potential impairment of a uniform heating of the target area, and the consequent risk of incomplete treatment.

Finally, by employing different solutions of encapsulation for the FBG sensors, it is possible to assess the impact of mechanical strain on their measurement and the level of resulting artifact. For example, Polito et al. developed in^79^ two FBG-based probes to compare thermal sensitivity and response time of a non-encapsulated FBG with those of two FBGs embedded within a metallic needle by implementing two different procedures (i.e., one was epoxy liquid encapsulated to maximize the stability with respect to the strain and the other by means of thermal paste to increment thermal conductivity between needle and optical fiber). They found a significant temperature overestimation for the encapsulated FBGs when positioned at small distances from the applicator, due to the metallic compound of the needles, but i) higher stability and temperature sensitivity in case of epoxy liquid encapsulation, and ii) low measurement error caused by simulated respiratory movements with respect to the non-encapsulated FBG.

Cross-sensitivity to strain caused by breathing has been investigated also in^83^ during LA of manually ventilated *ex vivo* swine lungs, comparing the recorded signal of encapsulated and bare FBGs and supporting temperature measurements with stereophotogrammetric Motion Capture system. Respiration artifact has been quantified and corrected by identifying the respiratory frequency and applying a stop-band filter to the recorded signals.

### Fiber Bragg gratings for microwave ablation monitoring

Like the previous paragraph, even for MWA a state-of-the-art collection about the use of FBG sensors to investigate the treatment outcomes from different perspectives is outlined in Table 4 and deepened in the following. Also for MWA one of the first works reporting on the use of FBGs dates to 2010, when Saxena et al.^108^ proposed a polymer coated array of ten FBGs placed in parallel to the applicator in a phantom approximating the muscle dielectric properties, comparing the FBG array performance with those of the Bowman probe. They found a good agreement between the temperature profiles recorded by the two probes and demonstrated the advantages of FBG arrays in such applications in terms of small size, multiplexing, stability, and spatial resolution.

**Table 4:** Key details of the main FBG-based temperature monitoring systems for MWA proposed in literature, organized by the addressed issue

Addressed issue	Tissue model	RF probe and treatment settings	Experimental configuration of FBG sensors	Reference
Feasibility of the use of FBG array for temperature monitoring and comparison with other thermometric solutions	Muscle equivalent phantom	Custom-made MW module *Model BSD 201* p = 10 W	1 array pf 10 FBGs 5 mm spaced, inserted inside a catheter in parallel to the applicator immediately close to it	Saxena et al.^108^
Experimental comparison of different MWA systems in terms of temperature profiles as the function of time and thermal damage observations	*Ex vivo* swine liver	- First tested MW module: Evident Ablation System, Covidien cooled-shaft antenna f = 915 MHz Power = 45 W Exposure time = 10 min - Second tested MW module: Emprint Ablation System, Covidien f = 2.45 GHz Power_1_ = 45 W Exposure time1 = 10 min Power_2_ = 100 W Exposure time2 = 4 min	2 FBGs 1 cm long inserted in parallel to the applicator at distances ranging from 10 mm to 30 mm	Saccomandi et al.^109^
Numerical and experimental evaluation of temperature profiles as the function of time and thermal damage	*Ex vivo* swine liver	Emprint Ablation System, Covidien f = 2.45 GHz Power = 100 W Exposure time = 4 min	8 FBGs at a distance from the antenna ranging from 1 cm to about 4.6 cm	Schena et al.^110^
Numerical and/or experimental analysis of blood perfusion contribution to the treatment outcomes	*Ex vivo* swine liver	Microwave Ablation System and SS-MWA-1531C Antenna, Surgnova Healthcare Technologies f = 2.45 GHz p = 50 W Exposure time = 8 min	3 arrays of 10 FBGs, each one 1 mm long and 2 mm spaced, perpendicular to the applicator, at distances ranging from 2 mm to 10 mm from it	Zaltieri et al.^111^
		Microwave Ablation System and SS-MWA-1531C Antenna, Surgnova Healthcare Technologies f = 2.45 GHz p = 75 W Exposure time = 8 min	4 arrays of 7 FBGs, each one 1 mm long and 2 mm spaced, in parallel to the applicator, at distances ranging from 3.3 mm to 29.1 mm from it	De Vita et al.^112^ De Vita et al.^113^
Experimental evaluation of the treatment enhancement induced by using NPs	*Ex vivo* swine liver	Custom-made MW module, *Leanfa Hybrid Generator* Solid brass applicator with a length of 16 cm and a diameter of 3 mm f = 2.45 GHz p = 60 W Exposure time = 2 min	3 arrays of 5 FBGs, each one 5 mm long and 10 mm spaced, in parallel to the applicator: one array immediately close to the applicator, one at 5 mm, and one at 10 mm from it	Jelbuldina et al.^114^
Experimental evaluation of the treatment effects on untraditional tissues	*Ex vivo* bovine femur and/or tibia	*Microwave Ablation System and SS-MWA-1531C Antenna, Surgnova Healthcare Technologies* f = 2.45 GHz p = 75 W Exposure time = 8 min	4 arrays of 10 FBGs, each one 1 mm long and 2 mm spaced, in parallel to the applicator, at distances ranging from 5 mm to 30 mm from it	De Tommasi et al.^115^ De Vita et al.^116^
	*Ex vivo* swine thyroid	*Certus 140TM and CertusPR, NeuWave Medical Technology* f = 2.45 GHz P1 = 20 W, P2 = 30 W Exposure time1 = 5 min Exposure time2 = 2.5 min	2 arrays of 10 FBGs, each one 1 mm long and 2 mm spaced, in parallel to the applicator, at distances ranging from 4.8 mm to 7.8 mm from it	De Vita et al.^117^

Saccomandi et al.^109^ evaluated the temperature profiles as the function of time recorded by means of two FBGs inserted at several distances from the applicator during the MW discharge by two different generators (i.e., 915 MHz vs. 2.45 GHz) in *ex vivo* swine livers. Under the same power and duration settings (i.e., 45 W for 10 min), they discovered higher temperatures and larger areas of ablation by applying the 2.45 GHz MW system. Moreover, by comparing their results with the ones previously obtained by Sun et al.,^118^ who, in contrast, employed several thermocouples at 5÷25 mm from the applicator and recorded significantly higher temperatures for the generator at 915 MHz, they have inferred that the ablation outcomes depend not only on generator frequency, but also on antenna design, cooling system, and control mechanism of the generator. Furthermore, the results obtained with the MW generator at 2.45 GHz have been compared with numerical simulations in,^110^ observing a Gaussian distribution for both theoretical and experimental temperature map inside the plane containing the applicator axis and the eight employed FBGs.

**Figure 13:**
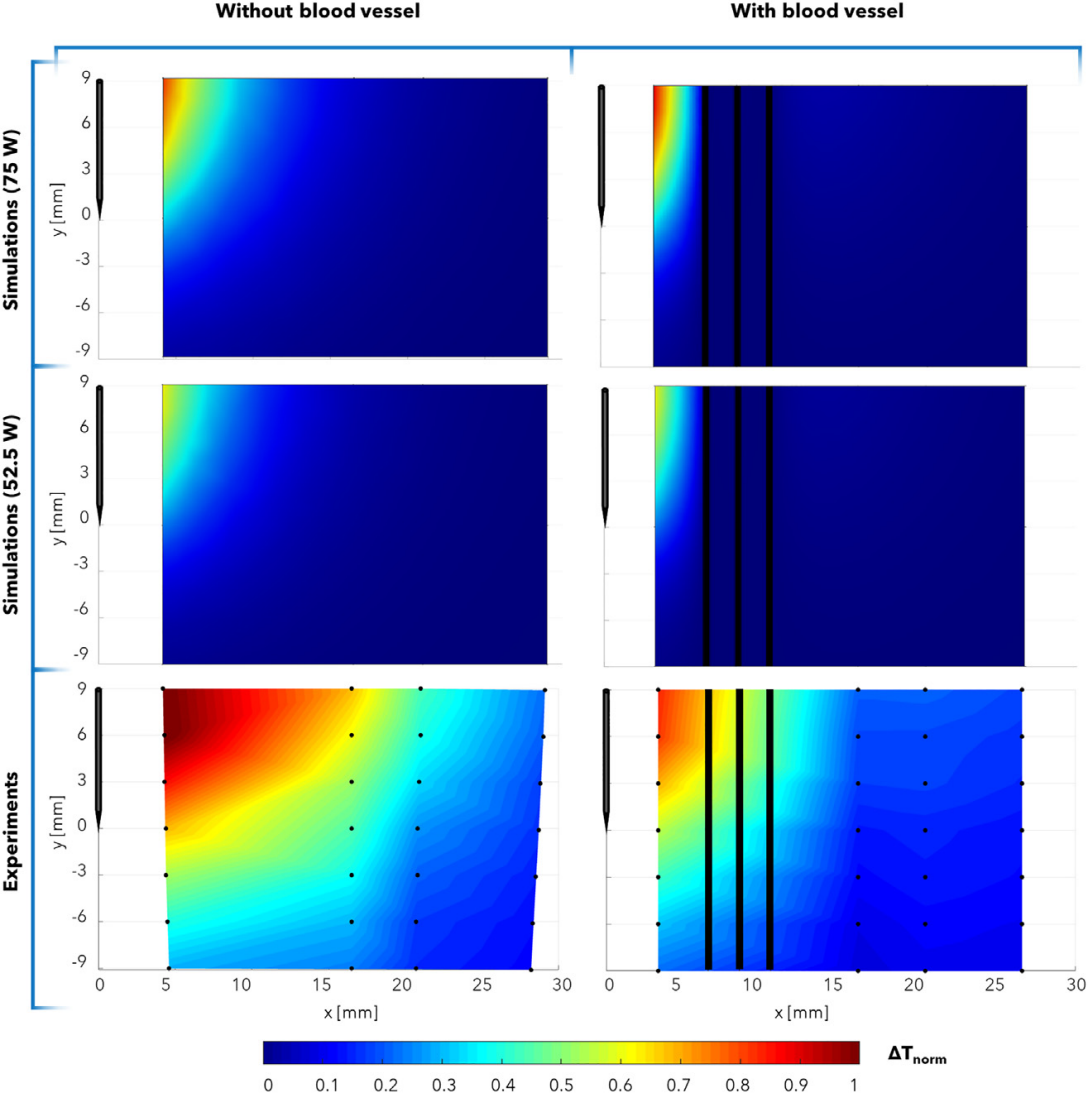
Examples of simulated and FBG-recorded temperature maps in the 2D space during MWA showing the blood vessel effect Simulated (first and second rows) and experimental (third row) maps of normalized ΔT at the end of an MWA test performed on *ex vivo* swine liver with (first column) and without (second column) blood vessel. The vertical black lines represent the vessel walls and axis, whereas the black dots in the FBG-recorded maps correspond to the experimental data. Reprinted with permission from.^113^ © IEEE.

It is worth noting that blood perfusion influence on MWA outcomes is not considered in the works discussed so far. In,^109^ for instance, at the end of each trial, authors checked if by chance some blood vessels were included into the ablated tissue and, if so, the trial was not included in the analysis. Indeed, although MWA mainly relies on direct heating rather than thermal conduction, becoming less dependent on heat sink effect due to blood perfusion, the presence of large blood vessel nearby the target region, together with the inherent inhomogeneities of the tissue, can limit the ability of MWA to create large necrotic lesions. For this reason, De Vita et al.,^112^^,^^113^ investigated the impact that a blood vessel nearby the applicator can have in terms of temperature trends and maps inside the target tissue from both numerical and experimental perspectives, implementing a perfusion system in *ex vivo* swine livers. By involving several arrays of FBGs strategically placed between applicator and blood vessel surrogate and beyond the vessel, they assessed and quantified a temperature drop in the presence of perfusion. The heat sink effect has been demonstrated by comparing simulated and experimental temperature profiles as the function of time at different distances from the applicator and, for the first time for an MW-ablated perfused organ, spatial maps of temperature were recorded to measure the different spatial extension of the thermal gradient nearby the applicator with and without vessel (see Figure 13). Similarly, in^111^ three arrays of ten FBGs each have been arranged in parallel to a vessel surrogate and perpendicularly to the applicator, at several distances from them, for testing MWA on perfused *ex vivo* swine liver, aiming to compare the recorded temperature trends and the respective time constants as the function of blood perfusion and distance from the applicator. The potential impairment of the MWA results caused by blood flow in the close vessels has led researchers to facilitate heat propagation toward the outer borders of the tumor by means of NPs injected into the target tissues, thus maximizing the volume of complete cell death. Jelbuldina et al.^114^ tested on *ex vivo* swine livers the feasibility of applying iron oxide magnetite NPs to enhance the efficiency of MWA, monitoring the results in terms of thermal maps as the function of time and space through three arrays of FBGs alongside the applicator, covering a measurement area of 10 mm × 45 mm and finding the highest extension of the ablated region and the fastest exceeding of the temperature threshold of 60°C when a density of 5 mg/mL of NPs was involved.

Using FBG-based measurement systems during MWA allowed to carry out innovative investigations of the effects when such recent thermal therapy is applied to untraditional organs, namely to tissues which usually undergo different thermal ablation treatments. This is the case of bone metastases, for which RFA still represents the most used thermal ablation treatment. However, the low conductivity and poor thermal conduction typical of bone tissue are limiting factors for RFA, recently encouraging the involvement of MWA. On this regard, in^115^^,^^116^ the experimental analysis of the temperature trends inside bovine bone tissue (i.e., femur and tibia) undergoing MWA has been assessed for the first time. By relying on a sensing network of 40 FBGs around the applicator, 3D maps of temperature within the target lesion are reported.

**Figure 14:**
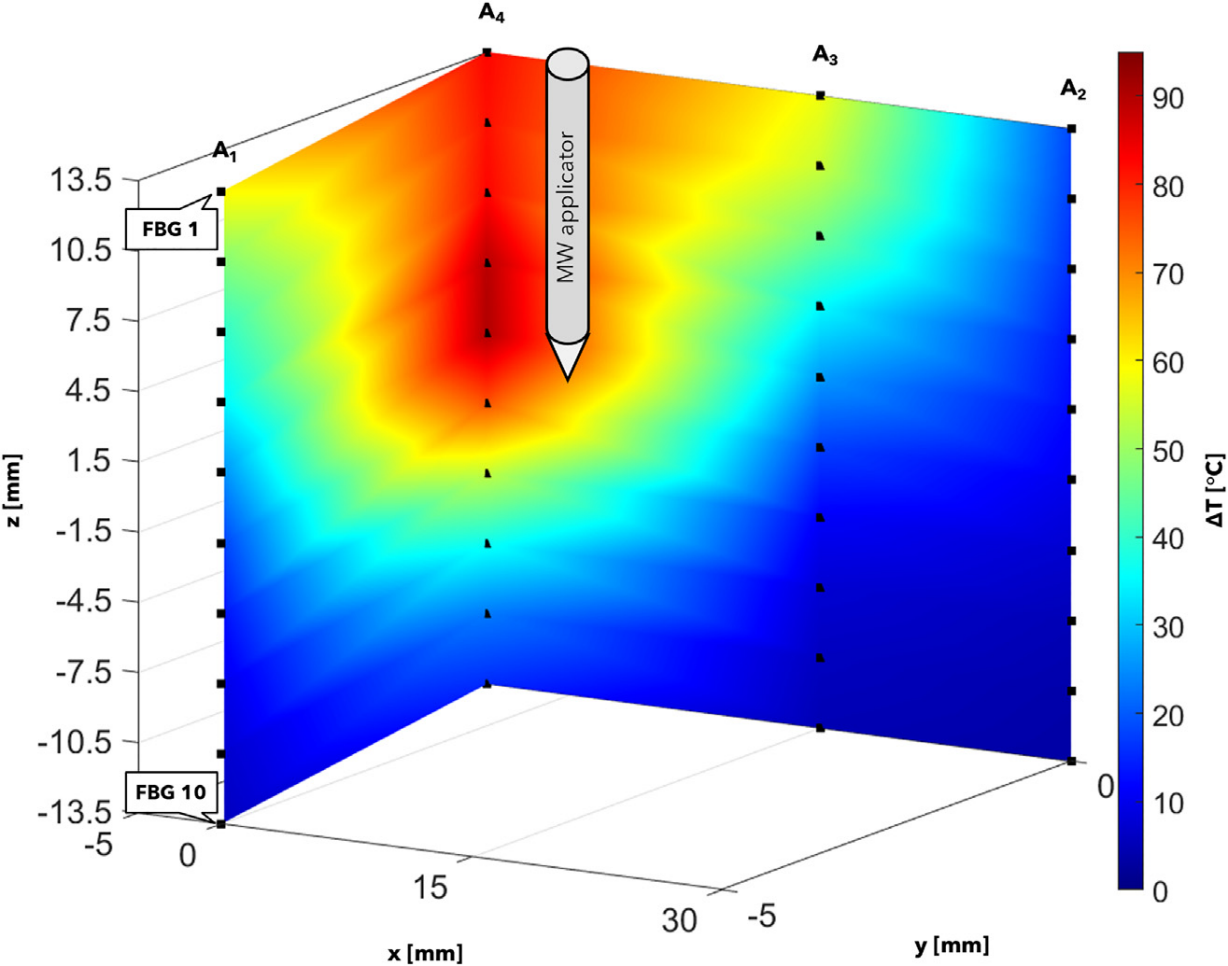
Example of FBG-recorded temperature map in the 3D space during MWA Three-dimensional map of ΔT recorded by forty FBGs at the end of an MWA test performed on *ex vivo* bovine femur. The axes are not to scale for the sake of clarity. Adapted from.^116^ Under a Creative Commons license.

The 3D thermal maps can provide immediate information on the spatial extension of the thermal gradient, its highest recorded value, and the spatial position of the maximum temperature (see Figure 14). Another innovative organ from the MWA point of view is thyroid, for which this kind of thermal treatment is gaining a newly increasing interest. In,^117^ the authors carried out a preliminary thermal analysis during MWA of thyroid samples by recording temperature trends and maps and comparing them for different settings of power and exposure time.

## Conclusion

The overall purpose of this work is to provide an overview of the current literature on the use of FBG sensors during RFA, LA, and MWA of *ex vivo* liver and other more challenging tissues. Such thermal ablation techniques have been described in terms of working principle, key features, benefits, and drawbacks with a view toward the comparison among them. The importance of thermal monitoring and mapping throughout any ablation treatment has been deepened, proving that temperature knowledge represents the successful strategy to provide feedback on the treatment outcomes and guide the ablation course toward a complete tumor treatment and a minimized recurrence. In other words, temperature monitoring addresses the crucial hurdle of such tumor treatments, i.e., the lack of ablation selectivity.

In this respect, the advantages of the FBGs for the temperature monitoring purposes and the pursuit of a controlled thermal ablation have been highlighted. Their multiplexing property, together with the flexibility, small size, fast response, CT- and MR-compatibility typical of the optical fibers, makes FBGs an optimal solution for a real-time minimally invasive monitoring aimed at controlling the treatment but also investigating the dependence of the ablation effects on factors such as tissue type and properties, thermal source, and blood perfusion.

In this framework, this work reviews the studies in literature on thermal ablation controlled by means of FBG sensors. For each investigated kind of ablation (i.e., RFA, LA, and MWA), such studies have been classified according to the specific issue addressed by the respective authors. Indeed, some of the reviewed articles focus on the experimental analysis of the treatment, others on the numerical versus experimental comparison, while still others deepen the influence of specific factors like the presence of blood vessels or use of NPs inside the target tissue on the ablation results.
